# A novel transcription factor CmMYB012 inhibits flavone and anthocyanin biosynthesis in response to high temperatures in chrysanthemum

**DOI:** 10.1038/s41438-021-00675-z

**Published:** 2021-12-01

**Authors:** Li-Jie Zhou, Zhiqiang Geng, Yuxi Wang, Yiguang Wang, Shenhui Liu, Chuwen Chen, Aiping Song, Jiafu Jiang, Sumei Chen, Fadi Chen

**Affiliations:** State Key Laboratory of Crop Genetics and Germplasm Enhancement, Key Laboratory of Landscaping, Ministry of Agriculture and Rural Affairs, Key Laboratory of Biology of Ornamental Plants in East China, National Forestry and Grassland Administration, College of Horticulture, Nanjing Agricultural University, Nanjing, Jiangsu 210095 China

**Keywords:** Secondary metabolism, Transcriptional regulatory elements

## Abstract

Flavones are among the major colorless pigments synthesized through branches of the flavonoid pathway in plants. However, due to the absence of a gene encoding flavone synthase (FNS) in the model plant *Arabidopsis thaliana* species, the regulatory mechanism of FNS-catalyzed flavone biosynthesis has rarely been studied in plants. Here, it was found that flavones play a predominant role in the elimination of excess reactive oxygen species (ROS) at high temperatures in colorless plant organs. A novel atypical subgroup 7 (SG7) R2R3-MYB transcription factor, CmMYB012, was found to be induced in response to prolonged high temperatures and to inhibit flavone biosynthesis by directly regulating *CmFNS*. Moreover, CmMYB012 was also found to inhibit anthocyanin biosynthesis by suppressing the expression of *CmCHS*, *CmDFR*, *CmANS*, and *CmUFGT*. *CmMYB012* overexpression exerted a negative influence on plant fitness and pink flower color formation, while *CmMYB012* suppression had the opposite effect in response to high temperatures. Our findings provide new insights into the mechanisms by which high temperatures regulate the metabolism of flavones and anthocyanins to affect plant fitness and flower color formation.

## Introduction

Flavonoids are mainly include flavones, flavonols, and anthocyanins and are distributed ubiquitously in a variety of plants. Among them, anthocyanins are colored pigments that confer colors to plant organs to attract pollinators for pollen transmission and to protect plants against abiotic stresses, such as UV radiation and drought^1–3^, while flavones and flavonols are colorless pigments integral to plant signaling and defense. Flavones contain C- or O-glycosylation and a hydroxylated B-ring, flavonols contain a 3-hydroxyflavone backbone, and both are the principal active substances that remove reactive oxygen species (ROS) in plants^4–6^.

Flavonoid biosynthesis in plants begins with the conversion of phenylalanine to p-coumaroyl-CoA, with L-phenylalanine ammonia-lyase (PAL) acting as the catalytic rate-limiting enzyme. Next, chalcone synthase (CHS) catalyzes the conversion of coumaroyl-CoA and malonyl-CoA to naringenin chalcone, which is the distinct step between the flavonoid metabolic pathway and other secondary metabolic pathways. Then, chalcone isomerase (CHI) catalyzes the conversion of naringenin chalcone to naringenin. Subsequently, flavanones (of which naringenin is one kind) are competitively catalyzed by FNS and flavanone 3-hydroxylase (F3H) to form flavones (mainly apigenin and luteolin) and dihydrokaempferol (DHK), respectively. Next, flavonol synthase (FLS) and dihydroflavonol 4-reductase (DFR) compete to catalyze the conversion of DHK to flavonols (mainly kaempferol and quercetin) and leucopelargonidin, respectively. Finally, leucopelargonidin forms anthocyanins under the catalysis of leucoanthocyanidin dioxygenase/anthocyanidin synthase (LDOX/ANS) and UDP-glucose: flavonoid 3-O-glucosyltransferase (UFGT)^7^. Interestingly, FNS and thus FNS-catalyzed flavone metabolism are absent in the model plant species *Arabidopsis thaliana*^8^. Recently, Ferreyra et al.^9^ reported that AtDMR6 in Arabidopsis has FNSI activity, but related studies are still scarce.

The biosynthesis of flavonoids is mostly regulated at the transcriptional level by the large MYB-bHLH-WD40 (MBW) complex, which is composed of an R2R3-MYB transcription factor (TF), a basic helix-loop-helix (bHLH) TF, and a WD40-repeat protein^10^. Among flavonoids, TFs related to anthocyanin metabolism have been extensively studied. For example, in *Arabidopsis*, R2R3-MYB TFs include MYB75 (also called PAP1), MYB90 (also called PAP2), MYB113, and MYB114; the bHLH TFs include TRANSPARENT TESTA 8 (TT8) and ENHANCER OF GLABRA 3 (EGL3); and the only WD40-repeat protein is TRANSPARENT TESTA GLABRA 1 (TTG1). These TFs have been shown to promote the expression of anthocyanin-associated structural genes such as *DFR* and *ANS*^11–13^. Conversely, another R2R3 MYB TF, MYB27, was found to inhibit anthocyanin biosynthesis by repressing the transcription of anthocyanin-associated genes^14^. Similarly, AtMYBL2, a protein with a single MYB domain, was found to negatively regulate anthocyanin biosynthesis in *Arabidopsis*^15^. In chrysanthemum (*Chrysanthemum* x *morifolium*), CmMYB6 and CmbHLH2 have been identified and found to interact with each other to enhance the expression of the *CmDFR* gene and ultimately promote the accumulation of anthocyanins^16^. For flavonols, it has been well established that SG7 MYB TFs containing the SG7 motif ([K/R][R/x][R/K]xGRT[S/x][R/G]xx[M/x]K) and the SG7-2 motif ([W/x][L/x]LS) at their C-termini, such as AtMYB11, AtMYB12, and AtMYB111, directly regulate flavonol biosynthesis-related genes such as *F3H* and *FLS* to affect the biosynthesis of flavonols^17–19^. For flavones, it was reported that GtMYBP3 and GtMYBP4 in gentian flowers and the MYB TFs P1 and P2 in maize positively regulate flavone biosynthesis^20,21^. Overall, TFs that negatively regulate flavone biosynthesis have rarely been studied in plants.

Flavonoid accumulation in plants is modulated by various environmental factors. Adverse environmental conditions such as excessive light, UV radiation, drought, and low temperature greatly promote the accumulation of flavonoids in plants^22–25^. An increase in flavonoids helps plants maintain normal growth and development by eliminating excess ROS produced under adverse conditions^26^. For example, MYB75 is phosphorylated and stabilized by MAP KINASE 4 (MPK4) under high-light conditions, thus promoting the expression of downstream anthocyanin-associated structural genes and anthocyanin accumulation in *Arabidopsis*^22^. Tobacco NtMYB12 directly activates the expression of *NtCHS* and *NtPT2*, leading to an increase in flavonol accumulation in response to low Pi stress^27^. High temperature is a common environmental stress that induces the generation of ROS in plants^28^; however, the accumulation of flavonoids decreases under high-temperature conditions. For example, it was found that the concentration of apple fruit anthocyanins decreased under hot temperatures^29^. In grape cultivation, high night temperatures generally reduce anthocyanin accumulation in berry skins^30^. One important reason is that genes encoding enzymes involved in the flavonoid biosynthesis pathway are inhibited by high temperatures, and some studies have been conducted to elucidate the related mechanisms. For example, a B-box TF, MdCOL4, is induced by high-temperature treatment to directly inhibit the transcription of *MdANS* and *MdUFGT*, leading to a decrease in anthocyanins in apple^31^. However, the mechanism by which high temperatures inhibit the accumulation of flavonoids other than anthocyanins, especially flavones, is still unclear.

Here, CmMYB012 was identified to regulate the high-temperature-inhibited accumulation of flavones and anthocyanins. CmMYB012 was further characterized as functioning in the inactivation of *CmFNS* and four other structural genes associated with anthocyanin biosynthesis at the transcriptional level. Finally, the mechanism by which CmMYB012 regulates flavone and anthocyanin biosynthesis under high-temperature conditions is summarized and discussed.

## Results

### Flavones are downregulated by prolonged high-temperature treatment

**Fig. 1:**
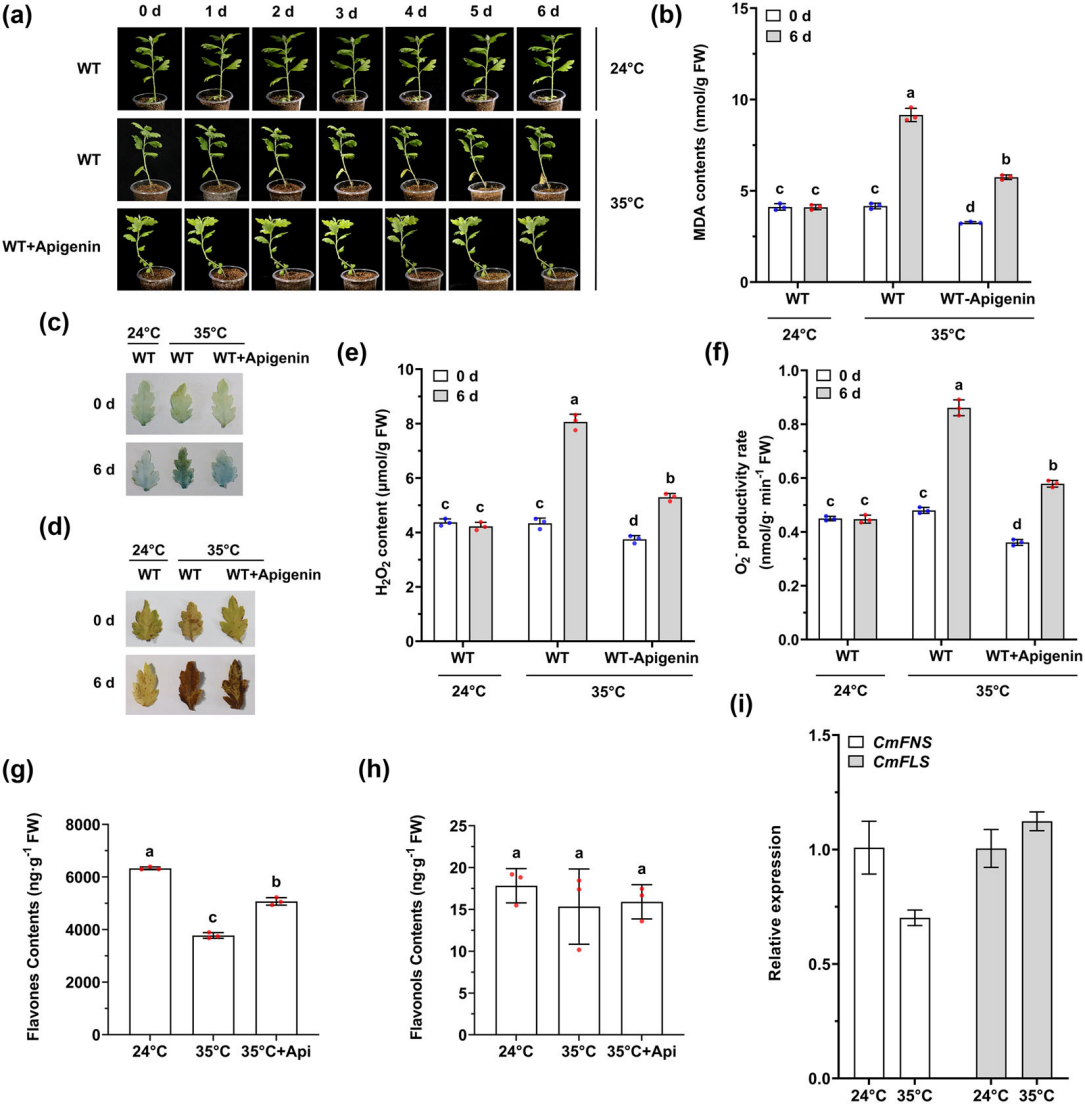
Flavones are downregulated by prolonged high-temperature treatment. **a** Phenotypes of plants subjected to high temperatures. **b** MDA contents in mature leaves. The error bars indicate the SDs of three biological replicates. The samples denoted by different letters are significantly different (*p* < 0.01, ANOVA, Tukey test). Each point represents one independent measurement. **c** Trypan blue staining for cell death in mature leaves. **d** DAB staining of O_2_^−^ and H_2_O_2_ in mature leaves. **e**, **f** H_2_O_2_ contents and O_2_^−^ productivity rates in mature leaves. The error bars indicate the SDs of three biological replicates. The samples denoted by different letters are significantly different (*p* < 0.01, ANOVA, Tukey test). Each point represents one independent measurement. **g**, **h** Flavone (apigenin and luteolin) and flavonol (kaempferol and quercetin) contents in mature leaves. The error bars indicate the SDs of three biological replicates. The samples denoted by different letters are significantly different (*p* < 0.01, ANOVA, Tukey test). Each point represents one independent measurement. Api indicates apigenin. (**i**) Relative expression of *CmFNS* and *CmFLS* in plants subjected to 24 °C and 35 °C for 6 days. The error bars indicate the SDs of three biological replicates

To explore the mechanism by which high temperatures affect plant fitness, 1-month-old cuttings of chrysanthemum (‘Fencui’ cultivar) were subjected to 35 °C for 6 days. Compared to the 24 °C control treatment, this treatment resulted in the phenotypes of wilting and withered leaves, especially for mature leaves near the base of the stem (Fig. 1a). Next, malondialdehyde (MDA) content measurements and detection of cell death in the leaves were conducted using spectrophotometry and trypan blue staining, respectively. The results showed that the MDA contents at 35 °C were much higher than those at 24 °C (Fig. 1b), and cell death, as indicated by strong staining, occurred throughout the mature leaves of the treated plants but not in those of the control plants (Fig. 1c), indicating that high temperatures caused damage to the cell membrane and caused cell death in the leaves. Previous studies have shown that excess ROS induced by abiotic stress cause significant damage to cell structures and lead to cell death^32^. To experimentally determine whether high temperatures caused excessive accumulation of ROS in leaves, histochemical staining with 3,3′-diaminobenzidine (DAB) was performed to detect ROS accumulation. As demonstrated in Fig. 1d, the leaves of plants subjected to 35 °C appeared to be much more deeply colored than the leaves of the controls did. Subsequently, the H_2_O_2_ content and the O_2_^−^ productivity rate were determined to further verify the generation of ROS. The results showed that the plants subjected to 35 °C generated more H_2_O_2_ and exhibited a higher O_2_^−^ productivity rate in their leaves than did the control plants (Fig. 1e, f), suggesting that high temperatures caused the accumulation of ROS in leaves. To address the question of whether the death of leaf cells at 35 °C was caused by the excessive accumulation of ROS, plants pretreated with apigenin, a flavone that can eliminate ROS, were subjected to 35 °C for 6 days. The results showed that due to the elimination of ROS, the MDA contents and cell death of leaves were much lower, and the plants exhibited greater resistance to high temperatures than did wild-type (WT) plants subjected to 35 °C (Fig. 1a–f).

In plants, flavonoids are proposed to act as ROS scavengers to maintain normal plant growth and development in response to abiotic stresses^26^. To determine the effect of high temperatures on flavonoids, flavones (apigenin and luteolin) and flavonols (kaempferol and quercetin) were identified via high-performance liquid chromatography (HPLC) analysis, and anthocyanin contents were measured using a spectrophotometer. The results showed that after treatment at 35 °C for 6 days, the average content of flavones in the leaves was 3774 ng/g FW, which was significantly lower than that in the control leaves (6330 ng/g FW) (Fig. 1g). However, the average content of flavonols in the leaves was maintained at a very low level, and there was no significant difference between the 24 °C (18 ng/g FW) and 35 °C (13 ng/g FW) treatments (Fig. 1h), while the anthocyanin content was too low to detect (Supplemental Fig. S1), suggesting that flavones played a predominant role in eliminating ROS and were suppressed by high-temperature treatment.

To investigate the mechanism by which flavones decreased under high-temperature conditions, RT-qPCR assays were conducted to measure the expression level of *CmFNS*. As a result, the expression of *CmFNS* dramatically decreased in plants after treatment at 35 °C for 6 days compared with that in the controls (Fig. 1i). In contrast, the transcription of *CmFLS* did not change significantly (Fig. 1i). These results indicated that prolonged high-temperature treatment suppressed *CmFNS* transcription.

### CmMYB012 directly binds to the AACATT element in the promoter of the CmFNS gene

**Fig. 2:**
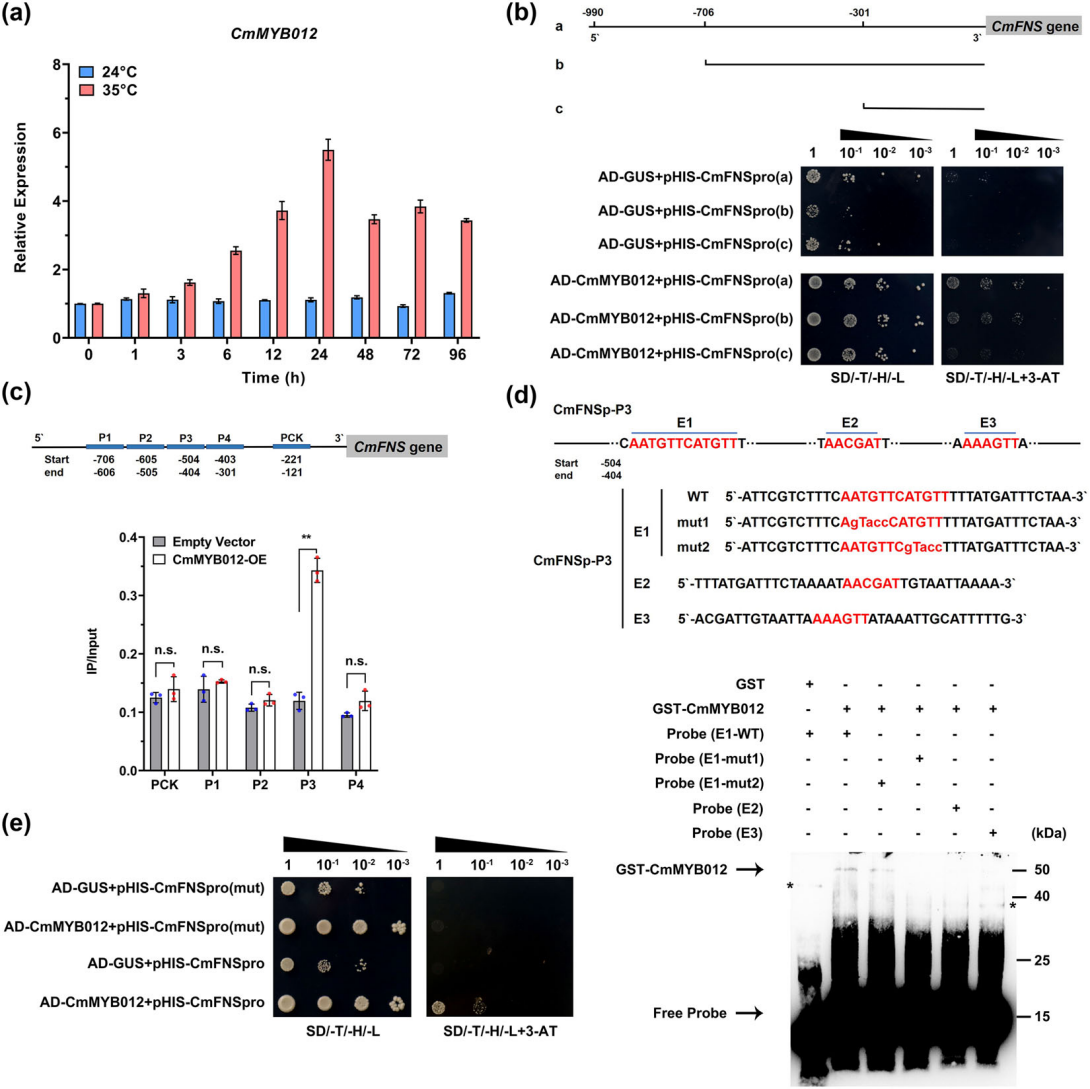
CmMYB012 directly binds to the AACATT element in the promoter of the *CmFNS* gene. **a** Relative expression of *CmMYB012* in plants subjected to 35 °C. Plants subjected to 24 °C were used as controls. The error bars indicate the SDs of three biological replicates. **b** Diagram of the *CmFNS* promoter, namely, from the start codon to a (−990 bp), b (−706 bp), and c (−301 bp), and interactions of CmMYB012 with these fragments in yeast cells. SD/-T/-H/-L indicates Trp, His, and Leu synthetic dropout media. A solution of 100 mM 3-AT was used. **c** ChIP-PCR analysis of the enrichment of DNA fragments P1 (−706 to −606 bp), P2 (−605 to −505 bp), P3 (−504 to −404 bp), and P4 (−403 to −301 bp) in the *CmFNS* promoter. The PCK fragment (−221 to −121 bp) was used as a negative control. The error bars indicate the SDs of three biological replicates. Significant differences are indicated with asterisks (*p* < 0.01, ANOVA, Tukey test). ‘n.s.’ indicates no significant difference. Each point represents one independent calculation. **d** EMSA of CmMYB012 binding to the P3 fragment. ‘WT’ indicates labeled DNA probes, while ‘mut’ indicates mutated probes. ‘+’ indicates presence, and ‘−’ indicates absence. The asterisks indicate nonspecific binding. **e** Interactions of CmMYB012 protein with the *CmFNS* promoter and its mutant version in yeast cells. SD/-T/-H/-L indicates Trp, His, and Leu synthetic dropout media. A solution of 100 mM 3-AT was used

To identify the potential regulators acting upstream of *CmFNS*, a 990 bp sequence of the *CmFNS* promoter was isolated and inserted into a *pHIS2* vector for yeast one-hybrid (Y1H) screening using a cDNA library of petal tissue of the ‘Fencui’ cultivar. As a result, a short cDNA fragment was obtained. The best-matched coding sequence of this fragment in chrysanthemum encodes an unidentified R2R3 MYB protein. This MYB protein was most closely related to the Pyrethrum TcMYB12-like gene but significantly differed in amino acid sequences at the C-terminal end (Supplemental Fig. S2a, b). We named this protein CmMYB012. RT-qPCR assays demonstrated that the transcription of *CmMYB012* was markedly induced by prolonged high-temperature treatment (Fig. 2a). Subsequently, the interaction between the promoter of *CmFNS* and the full-length CmMYB012 protein was verified with a Y1H assay. The results showed that, when *pHIS2-CmFNSpro* was coexpressed with *pGAD7-CmMYB012* in yeast, the strain was able to grow on plates of SD/-Trp/-His/-Leu media supplemented with 100 mM amitrole (3-AT). However, no growth was observed for the negative controls, in which *pHIS2-CmFNSpro* was coexpressed with *pGAD7-GUS* (Fig. 2b), indicating that CmMYB012 interacted with the promoter of *CmFNS* in yeast cells.

To examine the CmMYB012-binding region of the *CmFNS* promoter, the *CmFNS* promoter was divided into three fragments, a, b, and c, beginning from the start codon to 990, 706, and 301 bp upstream, respectively, and each fragment was inserted into a *pHIS2* vector. Y1H assays showed that CmMYB012 interacted with fragments a and b but not fragment c (Fig. 2b), indicating that the fragment from −301 to −706 bp was required for the promoter of *CmFNS* to interact with CmMYB012. To further verify the specific binding site of CmMYB012 to the *CmFNS* promoter, a *CmMYB012* overexpression vector was transformed into ‘Fencui’ chrysanthemum plants. RT-qPCR assays demonstrated that the transcript levels of *CmMYB012* increased in the two independent *OE-CmMYB012* (*35S:CmMYB012-GFP*) transgenic plants compared with the WT and the empty vector-transformed control (*35S:GFP*) plants (Supplemental Fig. S3). Subsequently, the fragment from −301 to −706 bp of the *CmFNS* promoter was further divided into four fragments, P1 (−606 to −706 bp), P2 (−505 to −605 bp), P3 (−404 to −504 bp), and P4 (−301 to −403 bp), to carry out chromatin immunoprecipitation (ChIP)-PCR assays. The results showed that fragment P3 was enriched in immunoprecipitates from transgenic plants overexpressing *CmMYB012-GFP* but not in those from empty vector-transformed plants, whereas fragments P1, P2, P4, and PCK (−121 to −221 bp of the promoter sequence was used as a negative control) were not enriched (Fig. 2c), indicating that CmMYB012 recognized fragment P3 in the *CmFNS* promoter. Wang et al.^33^ reported that R2R3-MYB TFs specifically recognize 5′-AACNDN-3′ (D: A, T or G) or 5′-ACC(A/T)A(A/C)-3′ elements. Next, the corresponding elements and their reverse-orientation versions were searched within fragment P3, and four candidate elements, AATGTTCATGTT (collectively named E1), AACGAT (E2), and AAAGTT (E3), were found (Fig. 2d). Subsequently, electrophoretic mobility shift assays (EMSAs) were conducted using a prokaryote-expressed and purified GST-CmMYB012 fusion protein. When an oligonucleotide containing an E1 sequence was used as a labeled probe, a specific DNA-CmMYB012 protein complex was detected, while E2 and E3 were not detected. Moreover, the DNA-CmMYB012 complex was still detectable when AATGTTCATGTT was mutated to AATGTTCgTacc, while the complex was not observed when AATGTTCATGTT was mutated to AgTaccCATGTT (Fig. 2d), indicating that the first 5′-AATGTT-3′ region within the E1 sequence was crucial for binding to CmMYB012 proteins. Finally, the Y1H assay showed that when the AACATT element (5′-AATGTT-3′ is its reverse complementary form) was mutated, the yeast strain in which *pHIS2-CmFNSpro* was coexpressed with *pGAD7-CmMYB012* was unable to grow on the selected media (Fig. 2e). Taken together, these results suggest that CmMYB012 specifically recognizes and binds to the AACATT element in the promoter of the *CmFNS* gene.

### CmMYB012 is an atypical SG7 R2R3-MYB protein

**Fig. 3:**
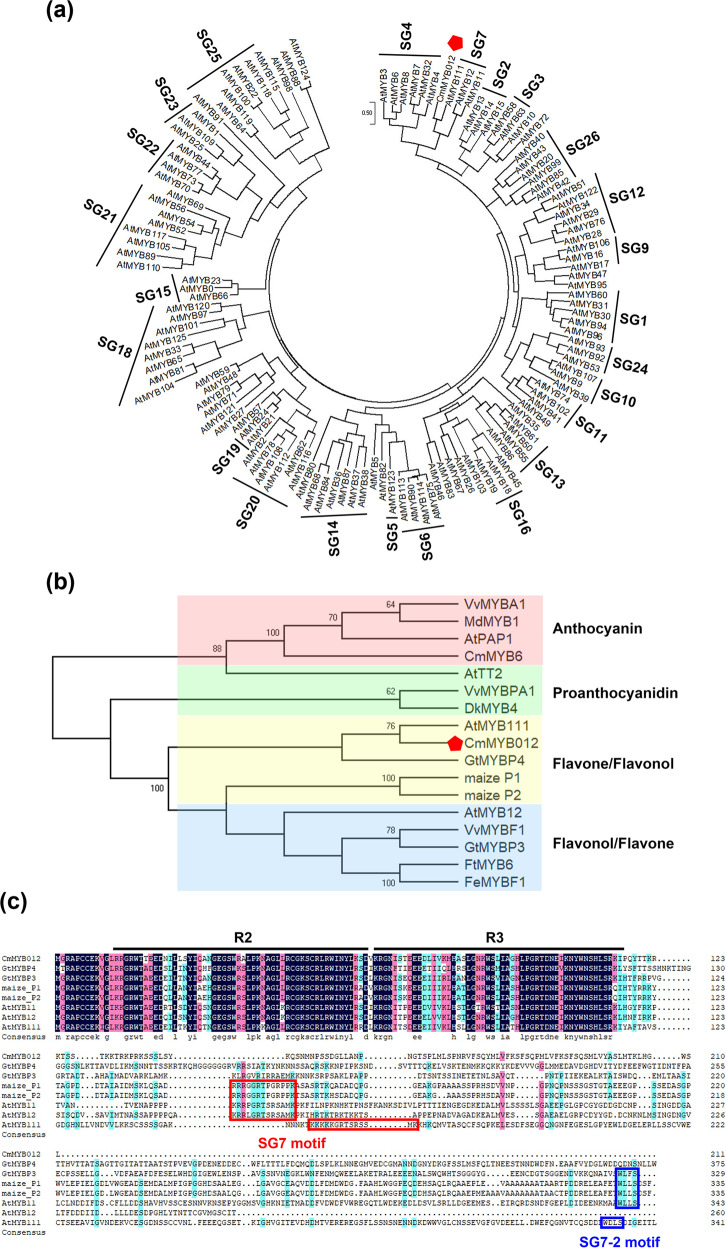
CmMYB012 is an atypical SG7 R2R3-MYB protein. **a** Phylogenetic relationships of CmMYB012 and R2R3 MYBs from Arabidopsis. A total of 126 protein sequences of the R2R3 MYBs in Arabidopsis were obtained from The Arabidopsis Information Resource (TAIR) database. The tree with the highest log likelihood (−79058.02) is shown. **b** Phylogenetic relationships of CmMYB012 and R2R3 MYBs from other species. The tree with the highest log likelihood (−10008.71) is shown. The percentage of trees in which the associated taxa clustered together is shown next to the branches. At, *Arabidopsis thaliana*; Cm, *Chrysanthemum morifolium*; Dk, *Diospyros kaki*; Fe, *Fagopyrum esculentum*; Ft, *Fagopyrum tataricum*; Gt, *Gentiana trifloral*; Md, *Malus domestica*; Vv, *Vitis vinifera*. **c** Sequence alignment of CmMYB012 and GtMYBP3, GtMYBP4, maize P1, maize P2, AtMYB11, AtMYB12, and AtMYB111 proteins. The R2 and R3 domains are indicated by the black lines above the sequences. The SG7 motif is indicated by the red lines around the sequences. The SG7-2 motif is indicated by the blue lines around the sequences

To analyze the phylogenetic relationships between CmMYB012 and the 126 R2R3 MYBs of Arabidopsis, a maximum-likelihood (ML) phylogenetic tree was constructed using MEGA X software. The results showed that CmMYB012 and three SG7 MYBs, AtMYB11, AtMYB12, and AtMYB111, formed a clade (Fig. 3a), indicating that CmMYB012 is an SG7 MYB protein. Generally, SG7 MYBs contain conserved SG7 and/or SG7-2 motifs at their C-termini and transcriptionally activate flavonol biosynthesis-related genes. However, transcriptional activity analysis showed that, unlike the three SG7 MYBs from Arabidopsis with transcriptional activation ability at their C-terminus, CmMYB012 had no transcriptional activation at either its N- or C-terminus (Supplemental Fig. S4a, b). We thus evaluated the phylogenetic relationships of CmMYB012 and 16 known MYB TFs related to the regulation of flavonoid biosynthesis in different species. The results showed that 17 MYB TFs could be classified into four groups [anthocyanins, proanthocyanidins, flavones/flavonols (mainly flavone), and flavonols/flavones (mainly flavonol)] based on their specific roles in regulating flavonoid biosynthesis. In addition to MYB TFs of Arabidopsis (e.g., AtMYB111 and AtMYB12), CmMYB012 was most closely phylogenetically related to GtMYBP4 and has been identified as playing a role in regulating flavone biosynthesis in gentian flowers (Fig. 3b). The protein alignment results showed that CmMYB012 had a conserved R2R3 MYB domain at the N-terminus but contained neither the SG7 motif nor the SG7-2 motif at its C-terminus. This case is similar to that of GtMYBP4 (Fig. 3c). Taken together, these results suggest that CmMYB012 is an atypical SG7 R2R3-MYB protein that is clustered together with other SG7 MYB proteins but does not contain a conserved SG7 and/or SG7-2 motif.

### CmMYB012 inactivates CmFNS transcription

**Fig. 4:**
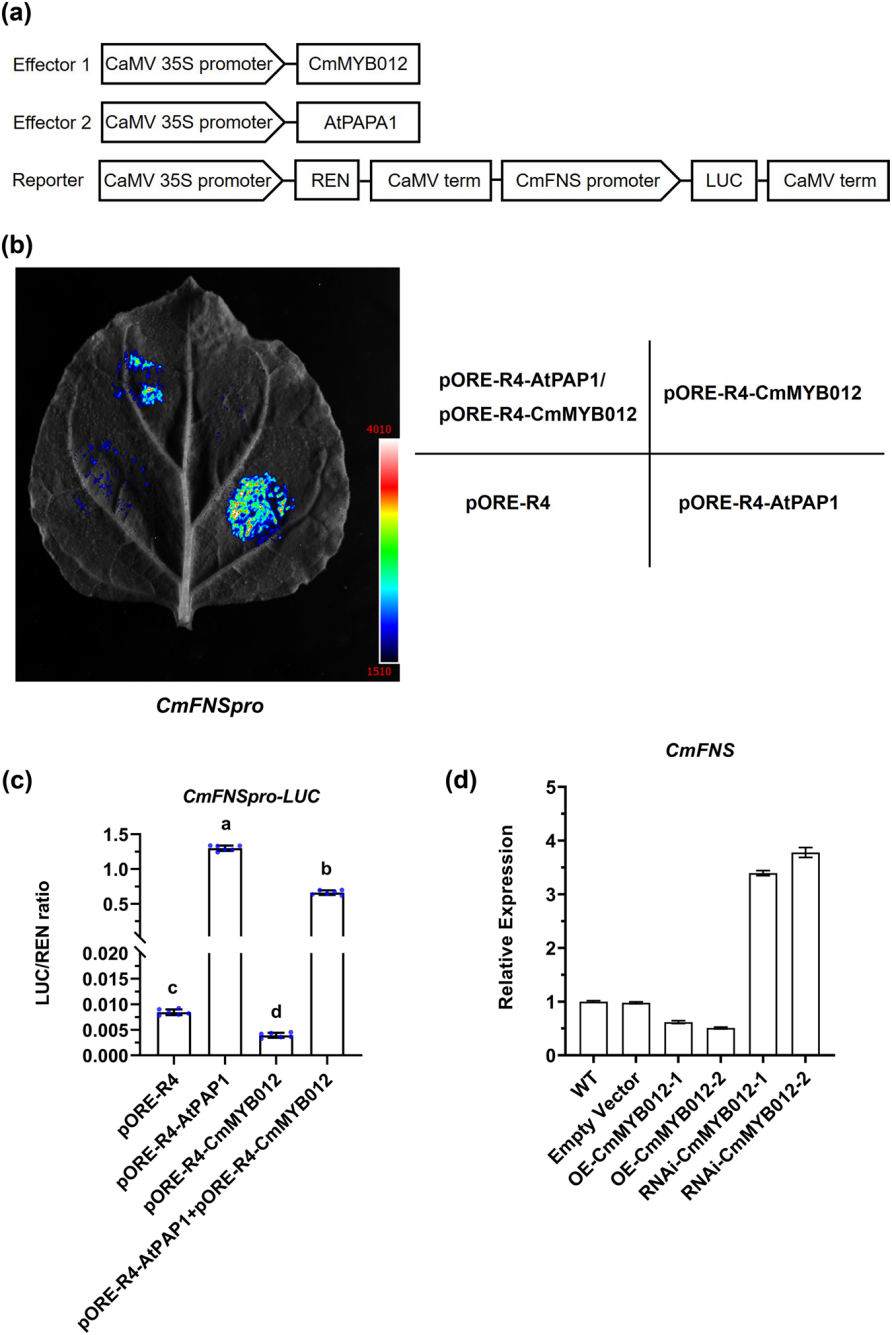
CmMYB012 inactivates *CmFNS* transcription. **a** Effector and reporter vector construction diagrams for dual-luciferase assays. **b** Graph showing the luminescence intensity. **c** Ratio of LUC to REN activity. The error bars indicate the SDs of six biological replicates. The samples denoted by different letters are significantly different (*p* < 0.01, ANOVA, Tukey test). Each point represents an independent calculation. **d** Relative expression of *CmFNS* in WT and transgenic chrysanthemum plants subjected to 24 °C. The error bars indicate the SDs of three biological replicates

Given that *CmFNS* transcription decreased as *CmMYB012* transcription peaked (Fig. 2a and Supplemental Fig. S1c), we hypothesized that CmMYB012 might act as a repressor of *CmFNS*. To test this hypothesis, a dual-luciferase assay was carried out in tobacco (*Nicotiana benthamiana*) leaves. The *CmFNS* promoter was fused to the *LUC* gene as a reporter. The *CmMYB012* effector construct was then expressed under the control of the *35S* promoter (*pORE*-*R4*-*CmMYB012*), and an *AtPAP1* effector (*pORE*-*R4*-*AtPAP1*) construct was used as a positive control (Fig. 4a). As demonstrated in Fig. 4b, the overexpression of *AtPAP1* dramatically increased the luminescence intensity around the injection site compared with that of the empty vector-transformed control, while coexpression of *CmMYB012* led to an obvious decrease in luminescence intensity. Moreover, the overexpression of *CmMYB012* alone decreased the luminescence intensity compared with that of the empty vector-transformed controls. The LUC/REN ratio was consistent with the luminescence intensity phenotype (Fig. 4c). Taken together, these results suggest that CmMYB012 inactivated the transcriptional activity of the *CmFNS* promoter.

Subsequently, RT-qPCR assays were conducted to assess the expression of *CmFNS* in WT, empty vector-transformed, *CmMYB012-OE* transgenic, and *RNAi-CmMYB012* transgenic chrysanthemum plants. The results showed that *CmFNS* transcription was significantly lower in the *CmMYB012-OE* chrysanthemum plants than in WT plants and empty vector-transformed control plants but higher in the *RNAi-CmMYB012* transgenic plants (Fig. 4d). These results further demonstrated that CmMYB012 acts as a repressor of *CmFNS* to inactivate *CmFNS* transcription.

### CmMYB012 negatively regulates plant fitness in response to high temperatures by inhibiting flavone biosynthesis

**Fig. 5:**
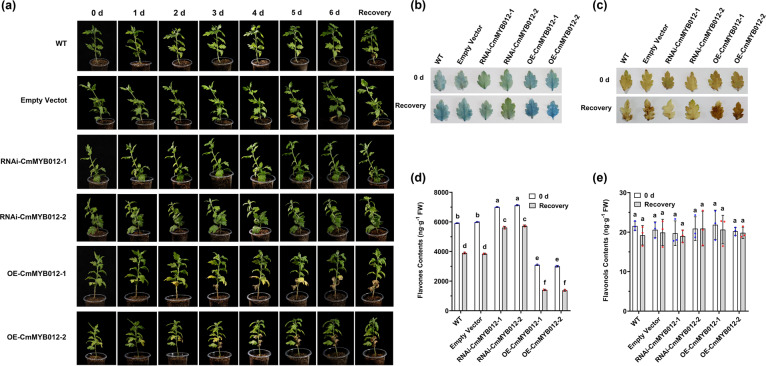
CmMYB012 negatively regulates plant fitness in response to high temperatures by inhibiting flavone biosynthesis. **a** Phenotypes of plants subjected to high temperatures. **b** Trypan blue staining for cell death in mature leaves. **c** DAB staining for O_2_^−^ and H_2_O_2_ in mature leaves. **d** Flavone (apigenin and luteolin) contents in mature leaves. The error bars indicate the SDs of three biological replicates. The samples denoted by different letters are significantly different (*p* < 0.01, ANOVA, Tukey test). Each point represents one independent measurement. **e** Flavonol (kaempferol and quercetin) contents in mature leaves. The error bars indicate the SDs of three biological replicates. The samples denoted by different letters are significantly different (*p* < 0.01, ANOVA, Tukey test). Each point represents one independent measurement

Based on the fact that CmMYB012 is induced in response to high temperatures and suppresses *CmFNS*, we deduced that CmMYB012 might inhibit flavone biosynthesis and negatively regulate plant fitness in response to high temperatures. To this effect, WT plants and two independent *OE-CmMYB012* and *RNAi-CmMYB012* transgenic lines were subjected to 35 °C for 6 days and then allowed to recover at 24 °C for one week, with empty vector-transformed plants used as controls. The results showed that, compared with the WT and the empty vector-transformed control plants, the *RNAi-CmMYB012* transgenic plants were more tolerant to high temperatures, as indicated by their overall better health, fewer dead cells and less ROS generation in their mature leaves, whereas the opposite effect was observed in the *OE-CmMYB012* transgenic plants (Fig. 5a–c; Supplemental Fig. S5a–c).

Subsequently, the contents of flavones and flavonols were analyzed via HPLC. The results showed that the average flavone content in the *RNAi-CmMYB012* transgenic plants was significantly higher than those in the WT plants and the empty vector-transformed control plants before treatment, while the contents in the *OE-CmMYB012* transgenic plants were significantly lower. After treatment at 35 °C for 6 days and one week of recovery, the WT and empty vector-transformed plants accumulated an average of 35% fewer flavones in their leaves than did those at 24 °C before treatment, while the contents of flavones in the *RNAi-CmMYB012* and *OE-CmMYB012* transgenic plants were 20% and 56%, respectively (Fig. 5d). However, there was no similar change in the amount of flavonols (Fig. 5e). These results indicated that CmMYB012 negatively regulates plant fitness in response to high temperatures by inhibiting flavone biosynthesis.

### CmMYB012 acts as a negative regulator of pink flower color formation

Given that *CmMYB012* was highly expressed in flower organs (Supplemental Fig. S4c), we suspected that CmMYB012 affects flower color formation. Thus, WT and three types of transgenic plants, namely, *OE-CmMYB012*, *RNAi-CmMYB012*, and empty vector-transformed control plants, were subjected to 24 °C and 35 °C at the budding stage to observe the flower color phenotype and evaluated the anthocyanin content after blooming.

**Fig. 6:**
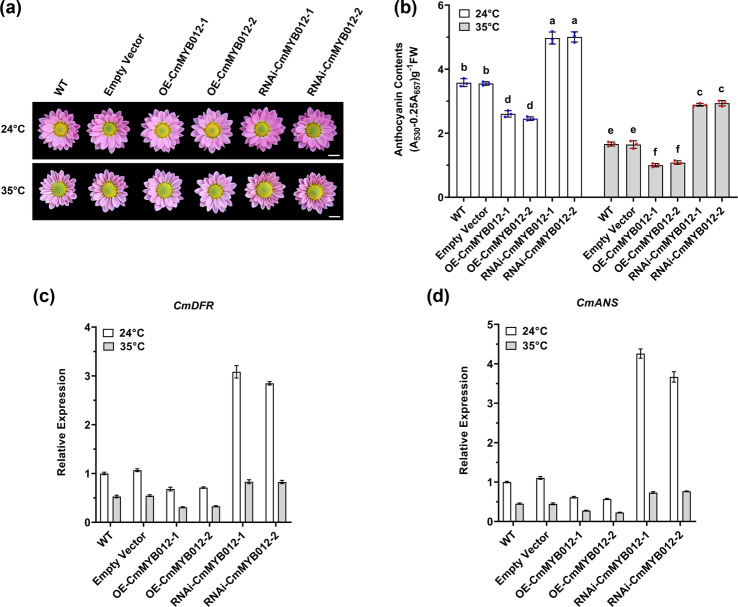
CmMYB012 acts as a negative regulator of pink flower color formation. **a** Flower color phenotypes of plants subjected to normal or high temperatures. **b** Anthocyanin contents in the petals of WT plants and transgenic plants subjected to 24 °C and 35 °C, respectively. The error bars indicate the SDs of three biological replicates. The samples denoted by different letters are significantly different (*p* < 0.01, ANOVA, Tukey test). Each point represents an independent measurement. **c**, **d** Relative expression of *CmDFR* and *CmANS* in the petals of WT and transgenic plants subjected to 24 °C and 35 °C, respectively. The error bars indicate the SDs of three biological replicates

The results showed that at 24 °C, the empty vector-transformed control plants and WT plants accumulated similar amounts of anthocyanins, suggesting that genetic transformation did not influence anthocyanin biosynthesis. In contrast, *CmMYB012* overexpression inhibited anthocyanin biosynthesis in the petals of transgenic plants, while its suppression promoted anthocyanin biosynthesis (Fig. 6a, b), indicating that CmMYB012 suppressed anthocyanin biosynthesis to inhibit pink flower color formation. Moreover, the anthocyanin contents in the WT plants and the empty vector-transformed control plants dramatically decreased at 35 °C, and the overexpression of *CmMYB012* exacerbated this phenomenon; however, the suppression of *CmMYB012* in the *RNAi-CmMYB012* transgenic plants partially restored the anthocyanin-based phenotype (Fig. 6a, b). The change in flavone content was consistent with that of anthocyanins (Supplemental Fig. S5d). Subsequently, the expression of anthocyanin-associated structural genes, such as *CmDFR* and *CmANS*, was measured via RT-qPCR. The results showed that the changes in the transcription of these genes were consistent with the anthocyanin phenotypes (Fig. 6c, d). Taken together, these results suggest that CmMYB012 acts as a negative regulator of both anthocyanin biosynthesis and pink flower color formation in chrysanthemum.

### CmMYB012 binds to the promoters of CmCHS, CmDFR, CmANS, and CmUFGT to inhibit their transcription

**Fig. 7:**
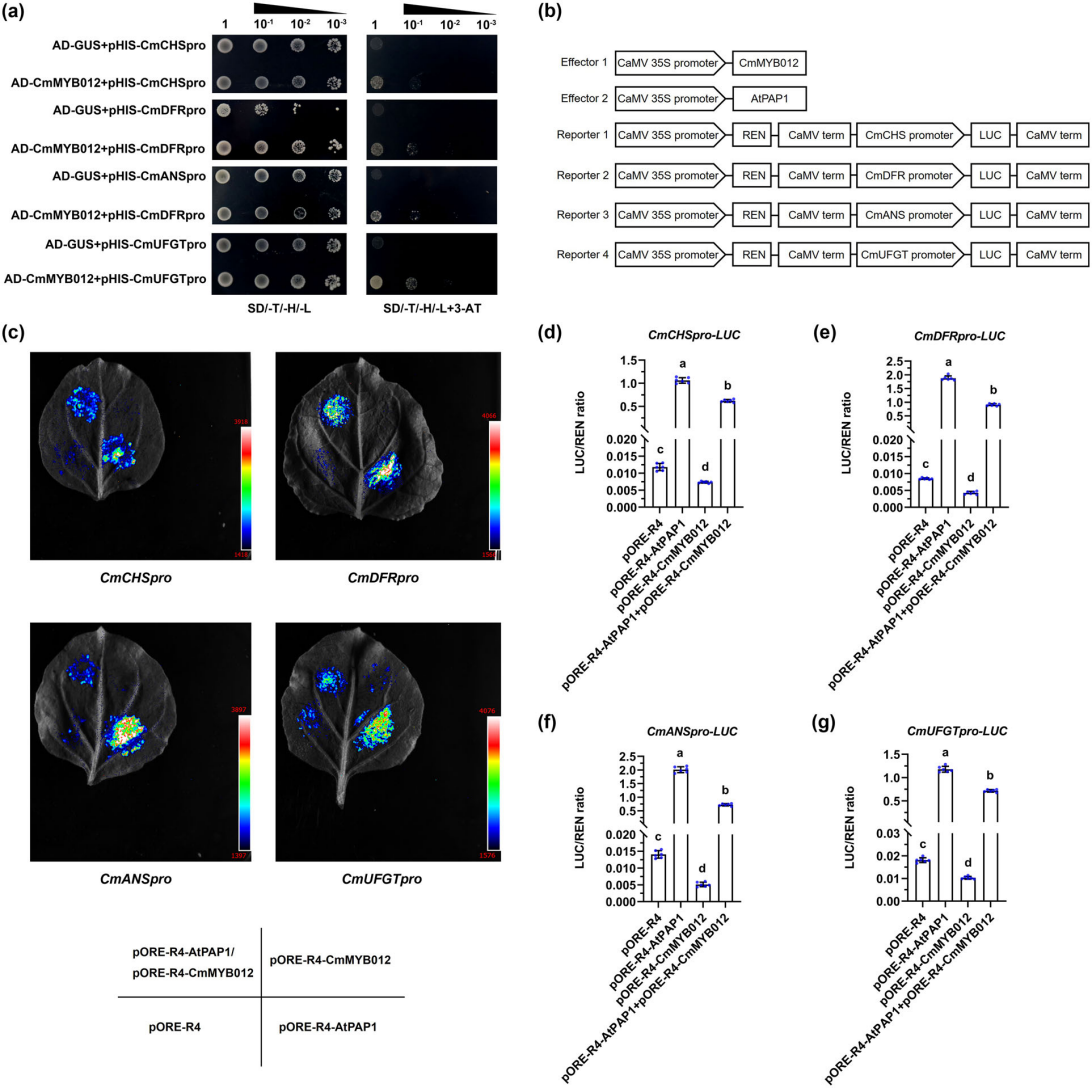
CmMYB012 binds to the promoters of *CmCHS*, *CmDFR*, *CmANS*, and *CmUFGT* to inhibit their transcription. **a** Interactions of CmMYB012 proteins and the promoters of *CmCHS*, *CmDFR*, *CmANS*, and *CmUFGT* in yeast cells. pGADT7-GUS was used as a negative control. SD/-T/-H/-L indicates Trp, His, and Leu synthetic dropout media. 3-AT concentrations: 100 mM for *CmCHSpro*, 50 mM for *CmDFRpro* and *CmANSpro*, and 80 mM for *CmUFGTpro*. **b** Diagrams of effector and reporter vector constructs for the dual-luciferase assays. (**c** Graph showing the luminescence intensity. **d**–**g** Ratio of LUC to REN activity. The error bars indicate the SDs of six biological replicates. The samples denoted by different letters are significantly different (*p* < 0.01, ANOVA, Tukey test). Each point represents an independent calculation

In plants, flavones and anthocyanins share the same biosynthesis pathway and compete with each other. The fact that CmMYB012 inhibits both flavones and anthocyanins prompted us to speculate that CmMYB012 might directly inhibit anthocyanin-associated structural genes. Thus, the promoter sequences of *CmCHS* (1021 bp), *CmCHI* (1379 bp), *CmF3H* (1041 bp), *CmFLS* (1090 bp), *CmDFR* (1826 bp), *CmANS* (1082 bp), and *CmUFGT* (1068 bp) were isolated to identify potential CmMYB012-recognized elements, specifically, AACATT sequences. As expected, AACATT elements were found in the promoters of *CmCHS*, *CmDFR*, *CmANS*, and *CmUFGT* (Supplemental Fig. S6a). Subsequently, Y1H assays were carried out to determine the interactions among CmMYB012 and the promoters of *CmCHS*, *CmDFR*, *CmANS*, and *CmUFGT*. The results showed that when *pHIS2-CmCHSpro*, *pHIS2-CmDFRpro*, *pHIS2-CmANSpro*, and *pHIS2-CmUFGTpro* fusion constructs were coexpressed with *pGAD7-CmMYB012* in yeast, the strains were able to grow on selective media; however, no growth was observed for the negative controls (Fig. 7a), indicating that CmMYB012 interacted with the promoters of *CmCHS*, *CmDFR*, *CmANS*, and *CmUFGT*.

Next, a dual-luciferase assay was carried out in tobacco leaves to determine whether CmMYB012 influences the transcriptional activity of the promoters of *CmCHS*, *CmDFR*, *CmANS*, and *CmUFGT*. The promoters of these four genes were subsequently fused to *LUC* genes to serve as reporters. The *CmMYB012* effector construct was expressed under the control of the *35S* promoter (*pORE-R4-CmMYB012)*, and the *AtPAP1* effector (*pORE-R4-AtPAP1*) construct was used as a positive control (Fig. 7b). The results showed that for each gene, the overexpression of *AtPAP1* dramatically increased the luminescence intensity around the injection site compared with that of the empty vector-transformed controls, while the coexpression of *CmMYB012* led to an obvious decrease in luminescence intensity. Moreover, the overexpression of *CmMYB012* alone decreased the luminescence intensity compared with that of the empty vector-transformed controls. In addition, the LUC/REN ratio was consistent with the luminescence intensity phenotype (Fig. 7d–g). These results suggest that CmMYB012 inactivated the transcriptional activity of the promoters of *CmCHS*, *CmDFR*, *CmANS*, and *CmUFGT*.

Moreover, RT-qPCR assays showed that the *CmCHS* and *CmUFGT* transcript accumulation was inhibited in the *CmMYB012-OE* plants but promoted in the *RNAi-CmMYB012* transgenic plants compared to the WT and empty vector-transformed control plants, and the suppression of both genes by high temperatures was partially restored by the silencing of *CmMYB012* but exacerbated by *CmMYB012* overexpression (Supplemental Fig. S6b, c). Combined with the *CmDFR* and *CmANS* expression data (Fig. 6c, d), these data suggest that CmMYB012 inhibits the transcription of *CmCHS*, *CmDFR*, *CmANS*, and *CmUFGT*.

## Discussion

In the background of global warming, high temperature is the principal abiotic stress that affects crop production^34^. In addition to the synthesis of heat- shock proteins, which act as molecular chaperones to prevent irreversible protein denaturation, the elimination of excess ROS by flavonoids is crucial for the ability of plants to cope with high-temperature stress^35^. In this study, it was found that flavones, not flavonols, are the main flavonoids that eliminate excess ROS at high temperatures in colorless plant organs. The decrease in flavones in response to prolonged high temperatures leads to stress symptoms in plants under long-term high-temperature conditions. Furthermore, a novel atypical SG7 R2R3-MYB TF, CmMYB012, was identified to regulate flavone biosynthesis through its ability to directly inhibit the transcription of the flavone synthesis-related gene *CmFNS*. The induction of *CmMYB012* is crucial for the downregulation of flavones under prolonged high temperatures.

RT-qPCR assays demonstrated that *CmFNS* was significantly upregulated under high-temperature treatment during the first 6 h but downregulated after 6 h and that its expression was lower than that in the 0-h control after 48 h. Moreover, there was no obvious pattern in the expression of *CmFLS* (Supplemental Fig. S1b, c), indicating that an unknown TF that positively regulates *CmFNS* in response to high temperatures may exist in plants. This is consistent with the concept that plants synthesize flavonoids to eliminate excess ROS after sensing ROS signals. Prolonged high-temperature treatment induced CmMYB012 to compete with an unknown TF to regulate the transcription of *CmFNS* and ultimately inhibited *CmFNS*. The results also showed that *CmMYB012* expression did not change significantly during the first 3 h after high-temperature treatment (Fig. 2a), suggesting that certain regulators may inactivate CmMYB012 under normal or short-term high-temperature conditions to maintain the normal mechanism of removing excess ROS in plants and that prolonged high-temperature treatment further passivates these regulators to release CmMYB012. These mechanisms may be an adaptive strategy for plants exposed to long-term high-temperature conditions, in which mature leaves are ultimately sacrificed to reduce transpiration under high-temperature conditions. In addition, RT-qPCR assays showed that in the *RNAi-CmMYB012* transgenic plants that were subjected to 35 °C, the transcript level of *CmFNS* decreased after 6 h but was maintained at a relatively high level afterward compared with that in the WT plants (Supplemental Fig. S1d), suggesting that prolonged high temperatures inhibit *CmFNS* in a partially CmMYB012-dependent manner.

Overexpression of *CmMYB012* led to a decrease in the accumulation of flavones, which weakened the ability of plants to cope with adverse environmental conditions, increasing plant sensitivity to stress (Fig. 5a–d). This is consistent with the finding that *CmMYB012* was expressed at a relatively low level in vegetative organs (Supplemental Fig. S4c). Higher levels of flavones in vegetative organs are beneficial for plants to cope with environmental changes during growth and development.

A reduction in anthocyanins under high temperatures is a common phenomenon in various plant species^29^^,^^30^^,^^36^. Some regulators have been found to play a role in this process. For example, ELONGATED HYPOCOTYL 5 (HY5) positively regulates anthocyanin biosynthesis by directly binding to the promoters of structural genes such as *CHS* and *F3H*^37^. In addition, the E3 ubiquitin ligase CONSTITUTIVE PHOTOMORPHOGENIC 1 (COP1) degrades HY5 to inhibit anthocyanin biosynthesis at high temperatures^38^. Here, CmMYB012 was found to directly inhibit anthocyanin biosynthesis in chrysanthemum flowers by suppressing the expression of *CmCHS*, *CmDFR*, *CmANS*, and *CmUFGT* at high temperatures (Fig. 6c, d, Supplemental Fig. S6b, c), improving the understanding of the mechanism by which high temperatures inhibit anthocyanin biosynthesis in plants. In addition, RT-qPCR assays showed that *CmF3H* did not respond to high temperatures, while *CmCHI* was significantly downregulated after high-temperature treatment (Supplemental Fig. S6d, e). Combined with the results showing that CmMYB012 did not regulate *CmCHI* (Supplemental Fig. S7b, d), these additional results suggest that high temperatures regulate *CmCHI* in a CmMYB012-independent manner.

Phylogenetic analysis showed that CmMYB012 was clustered together with other SG7 MYB proteins in Arabidopsis (Fig. 3a). In plants, SG7 MYBs have been reported to specifically activate flavonol biosynthesis-related genes such as *CHI*, *F3H*, and *FLS* to promote flavonol accumulation^17^. Based on our data, CmMYB012 did not directly bind to the promoters of *CmCHI*, *CmF3H*, and *CmFLS* and did not regulate their expression (Supplemental Fig. S7a–d). Moreover, the flavonol content did not change in either the *CmMYB012-OE* or the *RNAi-CmMYB012* transgenic plants (Fig. 5e), suggesting that CmMYB012 did not regulate flavonol biosynthesis. Combining the results that CmMYB012 did not perform transcriptional activation and did not contain the conserved SG7 and/or SG7-2 motifs at its C-terminal sequence (Fig. 3c, Supplemental Fig. S4a, b), we reason that CmMYB012 is a novel atypical SG7 R2R3-MYB protein that negatively regulates flavone biosynthesis.

The results from the assessment of tobacco showed that ectopic expression of *CmMYB012* resulted in a lower accumulation of anthocyanins in the flowers of these transgenic plants compared with the flowers of the WT plants, leading to a similar trend in flower color formation under both 24 °C and 35 °C conditions, as observed for the transgenic chrysanthemum flowers (Supplemental Fig. S8). Taken together, these results suggest that CmMYB012 acts as a negative regulator of anthocyanin biosynthesis and is conserved in plants.

In most cases, R2R3-MYB TFs, bHLH TFs, and WD40-repeat proteins form a large MBW complex to regulate structural genes involved in the flavonoid biosynthesis pathway at the transcriptional level^10^. Generally, when a MYB TF is expressed ectopically in other species, the simultaneous ectopic expression of a bHLH TF that interacts with it is necessary to produce an anthocyanin-related phenotype^39,40^. Here, the overexpression of *CmMYB012* alone in tobacco resulted in a decrease in anthocyanin contents in the flowers (Supplemental Fig. S8). There are two possibilities for this: (1) CmMYB012 is similar to AtMYB12 and can work alone without forming a complex with a bHLH TF^41^, or (2) the sequence of the bHLH TF that interacts with CmMYB012 in chrysanthemum is similar to that in tobacco, so CmMYB012 can interact with the corresponding bHLH protein in tobacco.

In plants, transcriptional repressors are divided into two types: passive and active repressors. The active repressors contain an ERF-associated amphiphilic repression (EAR) motif (F/LDLNxxP; F represents phenylalanine, L represents leucine, D represents aspartic acid, N represents asparagine, X represents any amino acid, and P represents proline) that interacts with corepressors such as TOPLESS (TPL) and AtSAP18 and function by altering the acetylation level at the transcription initiation site of downstream genes to suppress their expression^42,43^. However, the EAR motif sequence was not found in the amino acid sequence of CmMYB012, indicating that CmMYB012 may act as a passive repressor and function by competing with transcriptional activators for the DNA binding of downstream genes.

It is worth noting that after high-temperature treatment, the diameter of flower was also significantly smaller than that under normal temperatures (Supplemental Fig. S9). The overexpression or suppression of *CmMYB012* did not affect this trend, indicating that high temperatures may affect flower development in a CmMYB012-independent manner. In addition, although the contents of both flavones and anthocyanins were reduced, the flowers of the WT and transgenic plants did not wilt and wither like the leaves under high temperatures did (Fig. 6a). In plants, the presence of triplet chlorophyll and the electron transport chain in PSI and PSII makes chloroplasts major sites of ROS production^44^. The lack of chloroplasts in petals compared with leaves makes the former produce much less ROS at high temperatures, which is probably the main reason why flowers do not wilt and wither at high temperatures.

## Conclusions

**Fig. 8:**
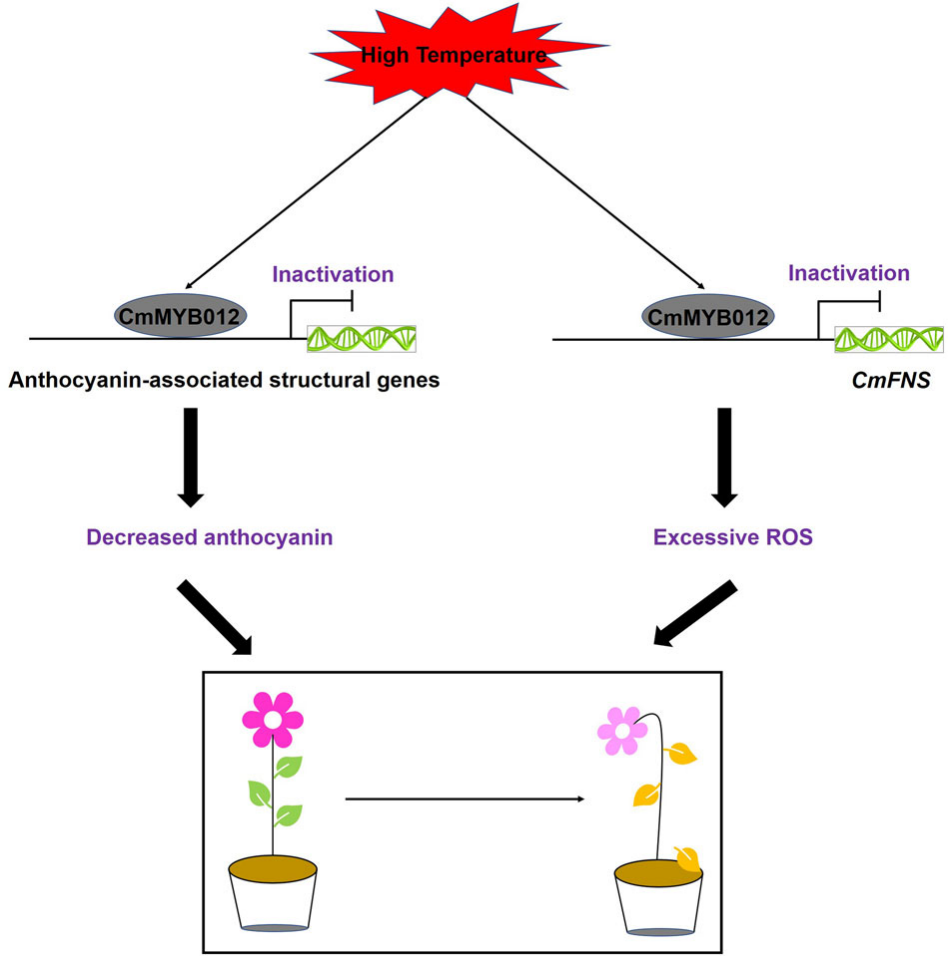
Model of CmMYB012-mediated chrysanthemum plant responses to high temperatures. High temperatures induce CmMYB012 to directly bind to the promoters of *CmFNS*, *CmCHS*, *CmDFR*, *CmANS*, and *CmUFGT* and inhibit their expression, leading to a decrease in flavones and anthocyanins. The reduction in flavones and anthocyanins weakens the ability of plants to cope with high-temperature stress and reduces the commercial value of chrysanthemum

In summary, our findings provide new insights into the mechanisms by which high temperatures regulate the biosynthesis of flavones and anthocyanins to affect plant fitness and pink flower color formation (Fig. 8). High temperatures cause CmMYB012 to directly bind to the promoters of *CmFNS*, *CmCHS*, *CmDFR*, *CmANS*, and *CmUFGT* and inhibit their expression, leading to a decrease in flavones and anthocyanins. The reduction in flavones and anthocyanins weakens the ability of plants to cope with high-temperature stress and reduces the commercial value of chrysanthemum. In our study, we elucidated the mechanisms by which high temperatures affect plant growth and development and provide a new target gene for the genetic engineering of increased crop yield and quality under high-temperature conditions.

## Materials and methods

### Plant materials and growth conditions

The chrysanthemum cultivar ‘Fencui’ was obtained from the Chrysanthemum Germplasm Resource Preservation Center, Nanjing Agricultural University, China. Cuttings were rooted in plug trays for 15 days, after which they were transplanted into cups and allowed to grow for one month under day/night temperatures of 24 °C/18 °C, under a 16/8 h light/dark photoperiod and a relative humidity of 70% for subsequent treatment. Tissue culture-generated ‘Fencui’ chrysanthemum plantlets were grown on Murashige and Skoog (MS) media supplemented with 2 mg/L 6-BA (6-benzylaminopurine) and 0.5 mg/L NAA (1-naphthylacetic acid) at 24 °C under a 16/8 h light/dark photoperiod. Early-flowering tobacco (*Nicotiana tabacum*) used for flower color analysis and tobacco (*Nicotiana benthamiana*) used for transient expression assays were grown under day/night temperatures of 24 °C/18 °C, a 16/8 h light/dark photoperiod and a relative humidity of 70%.

For high-temperature treatment, 1-month-old ‘Fencui’ chrysanthemum cuttings were subjected to 24 °C or 35 °C for 6 days for analysis. For the transgenic plants, 2-month-old WT and transgenic ‘Fencui’ chrysanthemum tissue cultures were transplanted into cups, allowed to grow for 28 days under the same conditions as the cutting seedlings were, subjected to 35 °C for 6 d, and then allowed to recover at 24 °C for one week for analysis. For flower color observation, cuttings of 2-month-old WT and transgenic tissue culture-generated plantlets were rooted in plug trays for 15 days and then cultivated in a greenhouse for 45 days at day/night temperatures of 24 °C/18 °C under a 16/8 h light/dark photoperiod and a relative humidity of 70%. Subsequently, cuttings from seedlings of similar size were continuously rooted in plug trays for 15 days, after which they were transplanted into flowerpots in a growth chamber and allowed to grow for 3 months at day/night temperatures of 24 °C/18 °C, an 8/16 h light/dark photoperiod, and a relative humidity of 70%. After flower buds appeared, the WT and transgenic plants were subjected to day/night temperatures of 35 °C/24 °C under an 8/16 h light/dark photoperiod and a relative humidity of 70% for high-temperature treatment; day/night temperatures of 24 °C/18 °C were used for control conditions. For high-temperature treatment, a cycle of 5 days of high temperature followed by 5 days of normal temperature was applied. The flower color phenotype was observed after blooming. For tobacco, WT and transgenic plants were subjected to an 8/16 h light/dark photoperiod and day/night temperatures of 35 °C/24 °C for the high-temperature treatment and day/night temperatures of 24 °C/18 °C for control conditions. The flower color phenotype was observed after blooming. Apigenin treatment was applied as described by Mekawy et al.^45^, with minor modifications. Briefly, the plants were sprayed evenly with 10 mg/L apigenin and allowed to dry for 6 h at 24 °C for high-temperature treatment.

### Vector construction and genetic transformation

For construction of the *35* *S:CmMYB012-GFP* vector, the full-length cDNA of *CmMYB012* was cloned into a *pORE-R4* vector under the control of the *35* *S* promoter. The *RNAi-CmMYB012* vector was constructed as described by Wang et al.^46^. The primer pairs used for the *RNAi-CmMYB012* vector construction are listed in Supplemental Table S1. Subsequently, the resultant vectors were genetically transformed into ‘Fencui’ chrysanthemum tissue cultures and early-flowering tobacco plants with *Agrobacterium tumefaciens* strain EHA105, as described by Simmons et al.^47^.

### Gene expression analysis

Total RNA was isolated from chrysanthemum plants and tobacco plants using a Quick RNA Isolation Kit (Huayueyang, Beijing, China) following the manufacturer’s instructions. First-strand cDNA was synthesized using HiScript II Q Select RT SuperMix for qPCR (Vazyme Biotech, Co., Ltd., Nanjing, China) according to the manufacturer’s instructions.

Next, the cDNA was diluted to 100 ng μL^−1^ with ddH_2_O for RT-qPCR, and the reactions were performed using TB Green® Premix Ex Taq™ II (Tli RNaseH Plus, Takara, Dalian, China) in a reaction volume of 20 μL. The following primer pairs were used: QM-F/QM-R for *CmMYB012*; QCS-F/QCS-R for *CmCHS*; QCI-F/QCI-R for *CmCHI*; QF3-F/QF3-R for *CmF3H*; QFN-F/QFN-R for *CmFNS*; QFL-F/QFL-R for *CmFLS*; QD-F/QD-R for *CmDFR*; QA-F/QA-R for *CmANS*; and QU-F/QU-R for *CmUFGT* (Supplemental Table S2). *CmEF1α* and *CmActin* were used as internal controls.

### Yeast one-hybrid assays

To carry out yeast (*Saccharomyces cerevisiae*) one-hybrid (Y1H) assays, full-length *CmMYB012* cDNA was inserted into a *pGADT7* vector, while the coding DNA sequence of *GUS* was inserted into a *pGADT7* vector as a negative control. Next, Promoter fragments of *CmFNS*, *CmCHS*, *CmDFR*, *CmANS*, *CmUFGT*, *CmCHI*, *CmF3H*, and *CmFLS* were cloned and subsequently inserted into *pHIS2* vectors. The primer pairs used for gene cloning are listed in Supplemental Table S1. Subsequently, all of the constructs were transformed into the *Y187* strain using the lithium acetate method. Subsequently, the transformed yeast cells were plated onto selective media lacking Trp, Leu, and His (SD/-Trp/-Leu/-His), and the colonies were then plated on -Trp/-Leu/-His media supplemented with an appropriate concentration of 3-AT and allowed to grow for 3 days at 28 °C.

### Electrophoretic mobility shift assays (EMSAs)

EMSAs were performed as described by An et al.^48^. Briefly, the *CmMYB012* coding sequence was cloned into a *PGEX-4T-1* vector for GST tag fusion. Next, GST-CmMYB012 proteins were expressed in and purified from *Escherichia coli* BL21 (DE3). 3′ biotin-labeled *CmFNS* promoter probes were used, which are shown in Fig. 2d. EMSAs were then carried out using a LightShift™ EMSA Optimization and Control Kit (Thermo, Shanghai, China) and Chemiluminescent Nucleic Acid Detection Module Kit (Thermo, Shanghai, China) following the manufacturer’s instructions.

### ChIP-PCR assays

ChIP-PCR assays were performed as described by An et al.^48^. Briefly, *35* *S:CmMYB012-GFP* transgenic chrysanthemum plants were subjected to ChIP-PCR assays using Pierce™ ChIP-grade Protein A/G Magnetic Beads (Thermo, Shanghai, China); *35* *S:GFP* transgenic chrysanthemum plants were used as controls. GFP recombinant rabbit monoclonal antibodies (Thermo, Shanghai, China) were used. Subsequently, the enriched DNA fragments were examined via RT-qPCR assays using the primers shown in Supplemental Table S3.

### Transient expression assays

Transient expression assays were performed as described by An et al.^48^. Briefly, promoter fragments of downstream genes such as *CmFNS* and *CmCHS* were cloned into a *pGreenII 0800-LUC* vector to generate reporter constructs. The coding sequences of *CmMYB012* and *AtPAP1* were cloned into a *pORE-R4* vector under the control of the *35S* promoter to generate effector constructs. The resultant vectors were subsequently transiently expressed in tobacco leaves (*Nicotiana benthamiana*). Luminescence was detected using an imaging apparatus designed for living organisms, and the ratio of LUC to REN activity was measured by an Infinite M200 luminometer (Tecan, Mannerdorf, Switzerland) and a Dual-Glo^®^ Luciferase Assay System (Promega).

### Histochemical staining analysis

To perform DAB staining, mature leaves of chrysanthemum plants were immersed in 1 mg/mL DAB dissolved in MES buffer (pH 5.5) for 12 h in the dark at room temperature. The leaves were then boiled in a 95% ethanol solution for 10 min for subsequent observations.

To perform trypan blue staining, mature leaves of chrysanthemum plants were immersed in trypan blue dye (0.02 g of trypan blue was dissolved in 10 mL of distilled water, after which this solution and 10 mL of phenol, 10 mL of glycerin, and 10 mL of lactic acid were mixed together and diluted with 95% ethanol to a 1:2 volume ratio before use) and boiled for 3 min. Subsequently, the leaves were discolored overnight in 2.5 g/mL chloral hydrate solution at room temperature for subsequent observations.

### MDA content, H_2_O_2_ content, and O_2_^−^ productivity rate measurements

To measure the MDA content, 0.5 g of leaves of chrysanthemum plants lacking a main leaf vein were homogenized and extracted using 5 mL of 0.05 M PBS (pH 7.8) at 4 °C. Next, the extract and 2.5 mL thiobarbituric acid were mixed together and then boiled for 15 min. After rapid cooling on ice and centrifugation at 4800 rpm for 10 min, spectrophotometric quantification of the supernatant at 450, 532, and 600 nm was performed to calculate the MDA content.

The H_2_O_2_ content was quantified as described by Gay and Gebicki.^49^, and the O_2_^−^ productivity rate was quantified as described by Shen et al.^50^.

### Flavone, flavonol, and anthocyanin content measurement

The flavone (apigenin and luteolin) and flavonol (kaempferol and quercetin) contents in the leaves and flowers of chrysanthemum plants were analyzed by HPLC at BioNovoGene (http://www.bionovogene.com/). Specifically, the analysis was carried out on a Waters ACQUITY UPLC instrument equipped with an AB 4000 Triple Quadrupole Mass Spectrometer (AB 4000). The samples were separated through an ACQUITY UPLC^®^ BEH C18 column (2.1 × 100 mm, 1.7 μm; Waters, America), and the column temperature was 40 °C. The mobile phases included solvent A (1–0.1% formic acid) and solvent B (100% methanol). The flow rate was 0.25 mL·min^−1^, and the injection volume was 5 μL. The mass spectrometry conditions included an electrospray ionization (ESI) source and negative ion ionization mode. The ion source temperature was 500 °C, the ion source voltage was −4500 V, the collision gas was 6 psi, the curtain gas was 30 psi, and both the atomization gas and the auxiliary gas were applied at 50 psi. Multiple reaction monitoring (MRM) was used for scanning. The samples were quantified according to the standard curves generated from the relative flavonoid components (Supplemental Table S4).

The total anthocyanin contents in the leaves and flowers of the chrysanthemum plants were quantified as described by Zheng et al.^51^.

### Accession numbers

Sequence data from this article can be found in the National Center for Biotechnology Information (NCBI) database. The genes and their accession number (listed in parentheses) are as follows: CmMYB012 (MW368976), CmCHS (MW368977), CmCHI (MW368978), CmF3H (MW368979), CmFNS (MW368980), CmFLS (MW368981), CmDFR (MW368982), CmANS (MW368983), and CmUFGT (MW368984).

## Supplementary information

Supplementary Material

## Data Availability

The authors confirm that all the experimental data are available and accessible via the main text and/or the supplemental data.
