# Supplementary material for: A novel transcription factor CmMYB012 inhibits flavone and anthocyanin biosynthesis in response to high temperatures in chrysanthemum
Source: Hortic Res. 2021 Dec 1;8:248. doi: 10.1038/s41438-021-00675-z (PMC8633327; doi:10.1038/s41438-021-00675-z)
Supplement: Supplementary file 1 — Supplementary Material [file 41438_2021_675_MOESM1_ESM.docx]

**A novel transcription factor CmMYB012 inhibits flavone and anthocyanin biosynthesis in response to high temperatures in Chrysanthemum**

Li-Jie Zhou, Zhiqiang Geng, Yuxi Wang, Yiguang Wang, Shenhui Liu, Chuwen Chen, Aiping Song, Jiafu Jiang, Sumei Chen, Fadi Chen*

State Key Laboratory of Crop Genetics and Germplasm Enhancement, Key Laboratory of Landscaping, Ministry of Agriculture and Rural Affairs, Key Laboratory of Biology of Ornamental Plants in East China, National Forestry and Grassland Administration, College of Horticulture, Nanjing Agricultural University, Nanjing, Jiangsu 210095, China.


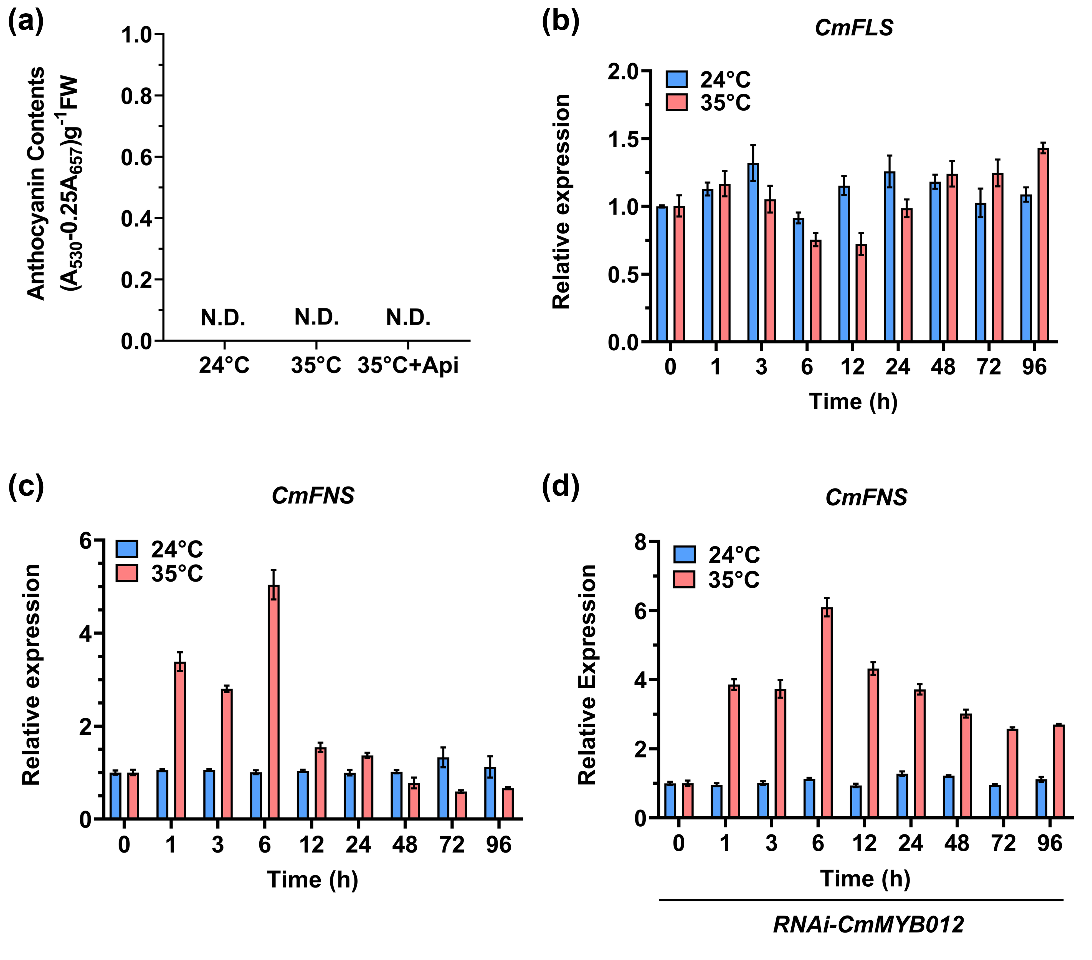


**Supplemental Figure S1.** **Anthocyanin contents and gene expression changes in chrysanthemum plants in response to high temperatures**

(a). Anthocyanin content in the mature leaves. N.D. indicates not detected. Api indicates apigenin. (b) and (c). Relative expression of *CmFLS* and *CmFNS* in plants at 35 °C. The plants treated at 24 °C were used as controls. The Error bars indicate the SDs for three biological replicates. (d). Relative expression of *CmFNS* in the *RNAi-CmMYB012* transgenic plants at 35 ℃. The transgenic plants treated at 24 ℃ were used as controls. The Error bars indicate the SDs for three biological replicates.


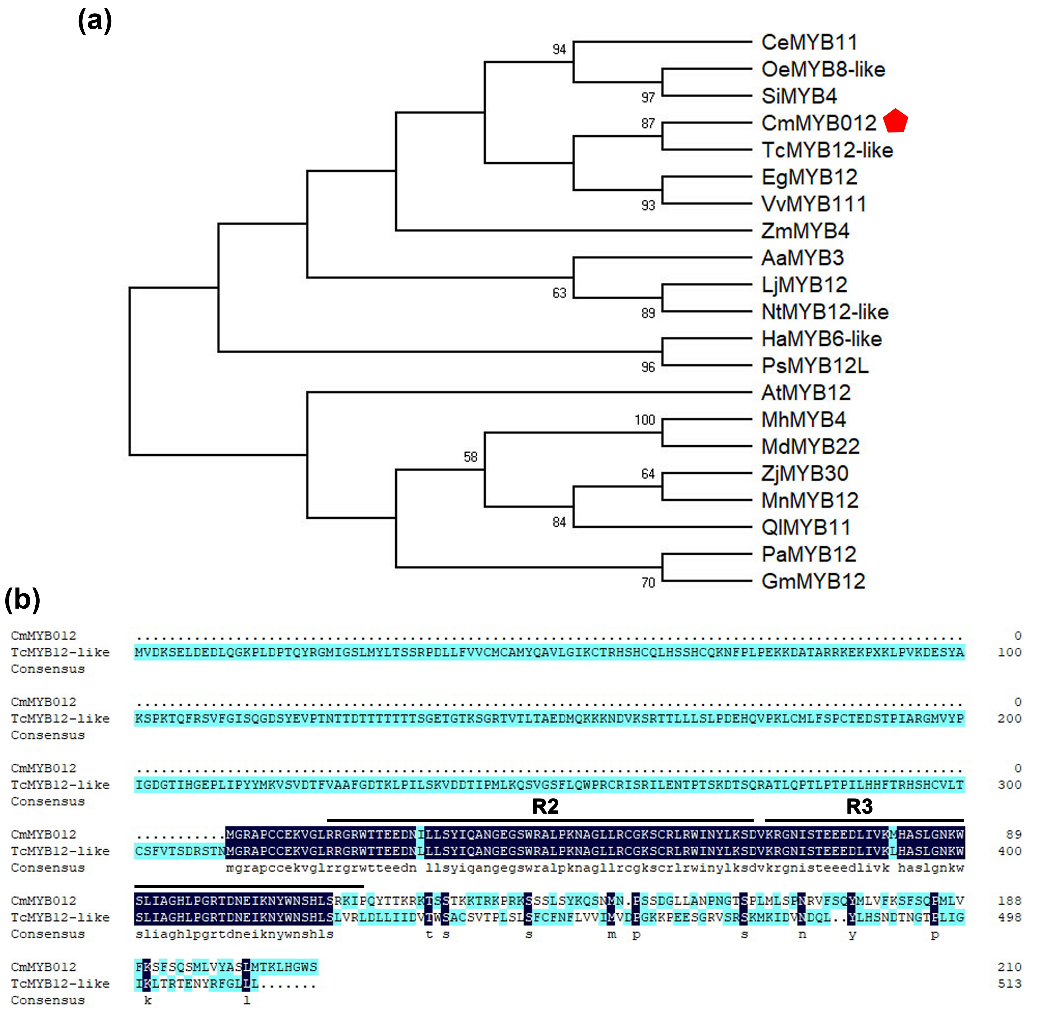


**Supplemental Figure S2. Phylogenetic relationships of CmMYB012 homologous proteins in twenty plant species**

(a). Twenty homologous protein sequences of the CmMYB012 protein in different plant species were obtained from the NCBI database. The tree with the highest log likelihood (-15117.22) is shown. The percentage of trees in which the associated taxa clustered together is shown next to the branches. Aa, *Artemisia annua*; At, *Arabidopsis thaliana*; Ce, *Coffea eugenioides*; Cm, *Chrysanthemum morifolium*; Eg, *Eucalyptus grandis*; Gm, *Glycine max*; Ha, *Helianthus annuus*; Lj, *Lonicera japonica*; Md, *Malus domestica*; Mh, *Malus hybrid*; Mn, *Morus notabilis*; Nt, *Nicotiana tabacum*; Oe, *Olea europaea*; Pa, *Prosopis alba*; Ps, *Paeonia suffruticosa*; Ql, *Quercus lobata*; Si, *Sesamum indicum*; Tc, *Tanacetum cinerariifolium*; Vv, *Vitis vinifera*; Zj, *Ziziphus jujuba*; Zm, *Zea mays*. (b). Sequence alignment of CmMYB012 and TcMYB12-like proteins. The R2 and R3 domain are indicated by the black lines above the sequences.


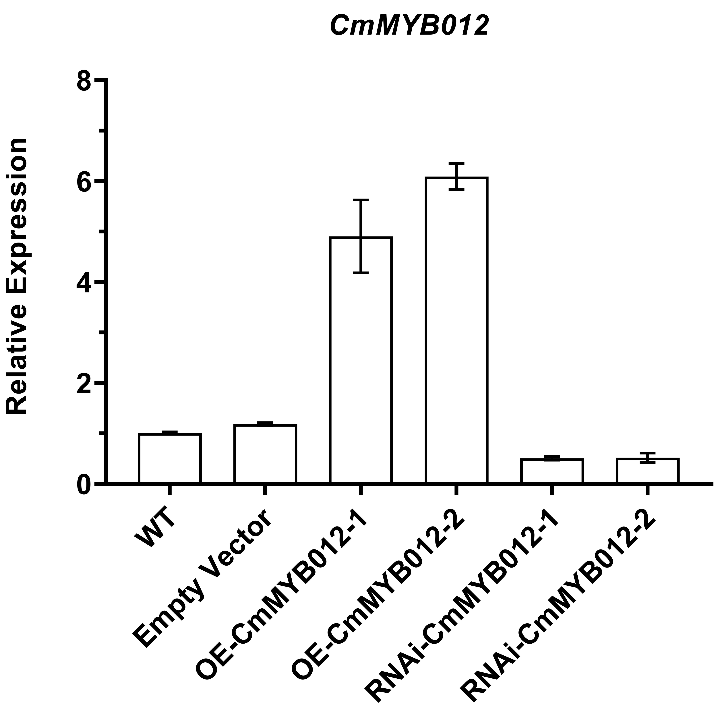


**Supplemental Figure S3. Identification of transgenic chrysanthemum plants**

Relative expression of *CmMYB012* in WT, the *Empty Vector* (*35S:GFP*), the *OE-CmMYB012* (*35S:CmMYB012-GFP*), and the *RNAi-CmMYB012* transgenic chrysanthemum plants. The Error bars indicate the SDs for three biological replicates.


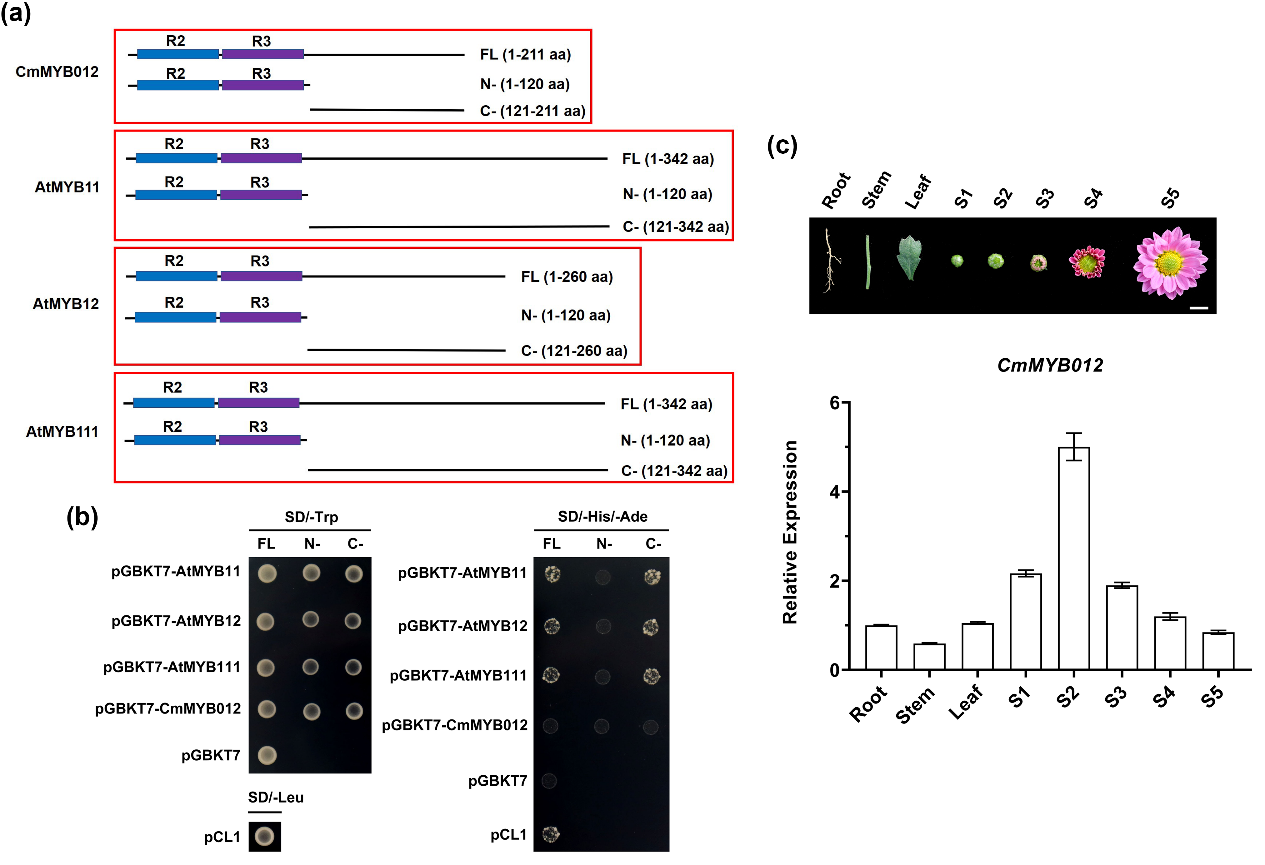


**Supplemental Figure S4. Transcriptional activity and expression pattern analysis of CmMYB012**

(a). Diagrams of the CmMYB012, AtMYB11, AtMYB12, and AtMYB111 protein sequences. FL, full length. N-, N-terminal. C-, C-terminal. (b). Transcriptional activity analysis of CmMYB012, AtMYB11, AtMYB12, and AtMYB111. pCL1 was used as a positive control and the empty vector pGBKT7 was used as a negative control. FL, full length. N-, N-terminal. C-, C-terminal. (c). The expression pattern of *CmMYB012* in different tissues and flowering processes (S1: flower bud, S2: broken bud, S3: dew color, S4: first bloom, and S5: full bloom) of ‘Fencui’ chrysanthemum cultivar. In particular, petals of different flowering processes were collected to detect the expression of *CmMYB012*. The Error bars indicate the SDs for three biological replicates. Scale bars = 1 cm.


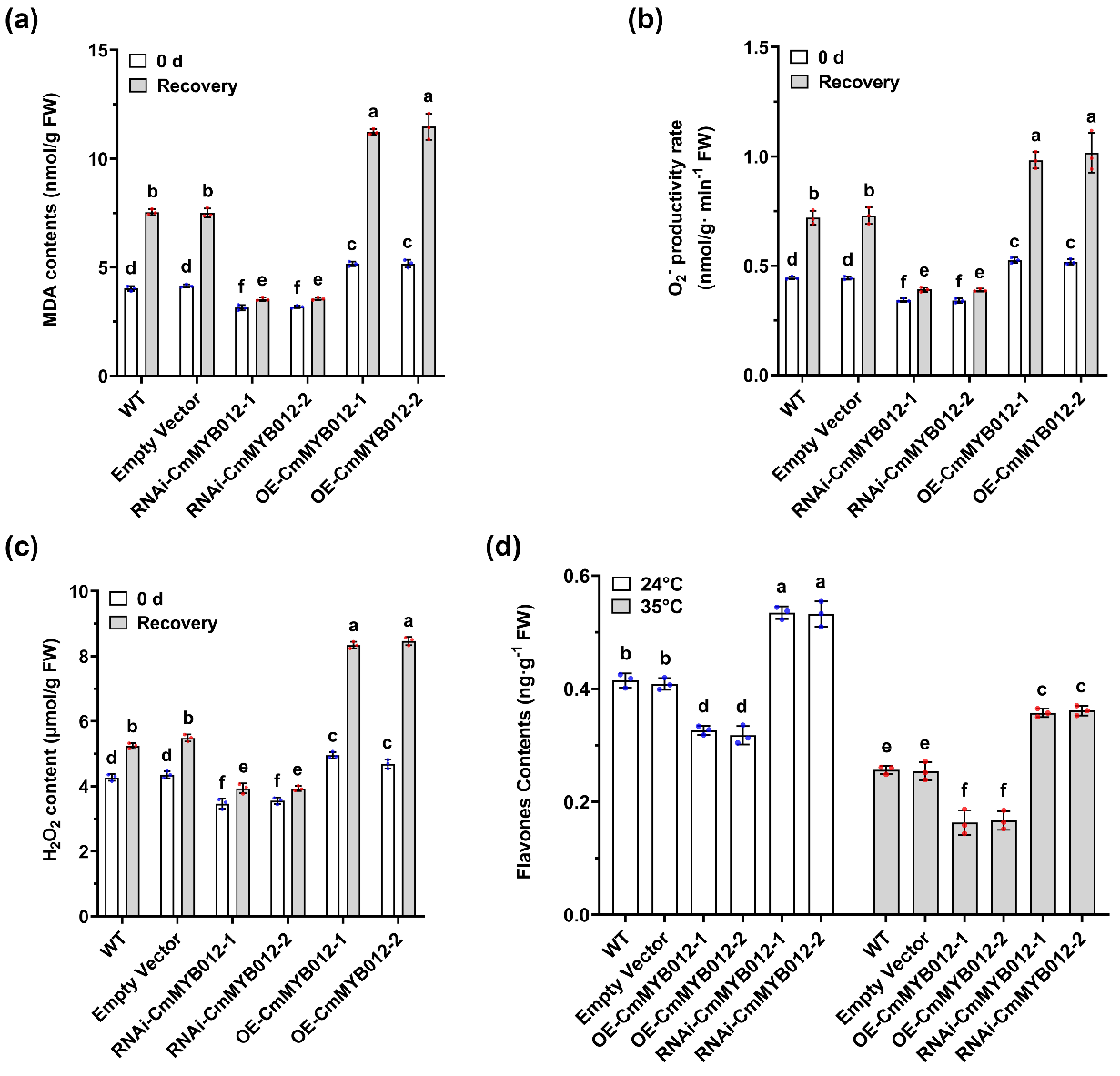


**Supplemental Figure S5. MDA content, O_2_^-^ productivity rate, H_2_O_2_ content and flavone content changes in transgenic chrysanthemum plants in response to high temperatures**

(a). MDA content in the mature leaves. The Error bars indicate the SDs for three biological replicates. Samples denoted by different letters are significantly different (p < 0.01, ANOVA, Tukey correction). Points represent each independent measurement. (b) and (c). O_2_^-^ productivity rate and H_2_O_2_ content in the mature leaves. The Error bars indicate the SDs for three biological replicates. Samples denoted by different letters are significantly different (p < 0.01, ANOVA, Tukey correction). Points represent each independent measurement. (d). Flavone content in petals of WT and the transgenic plants treated at 24 ℃ and 35 ℃, respectively. The Error bars indicate the SDs for three biological replicates. Samples denoted by different letters are significantly different (p < 0.01, ANOVA, Tukey correction). Points represent each independent measurement.


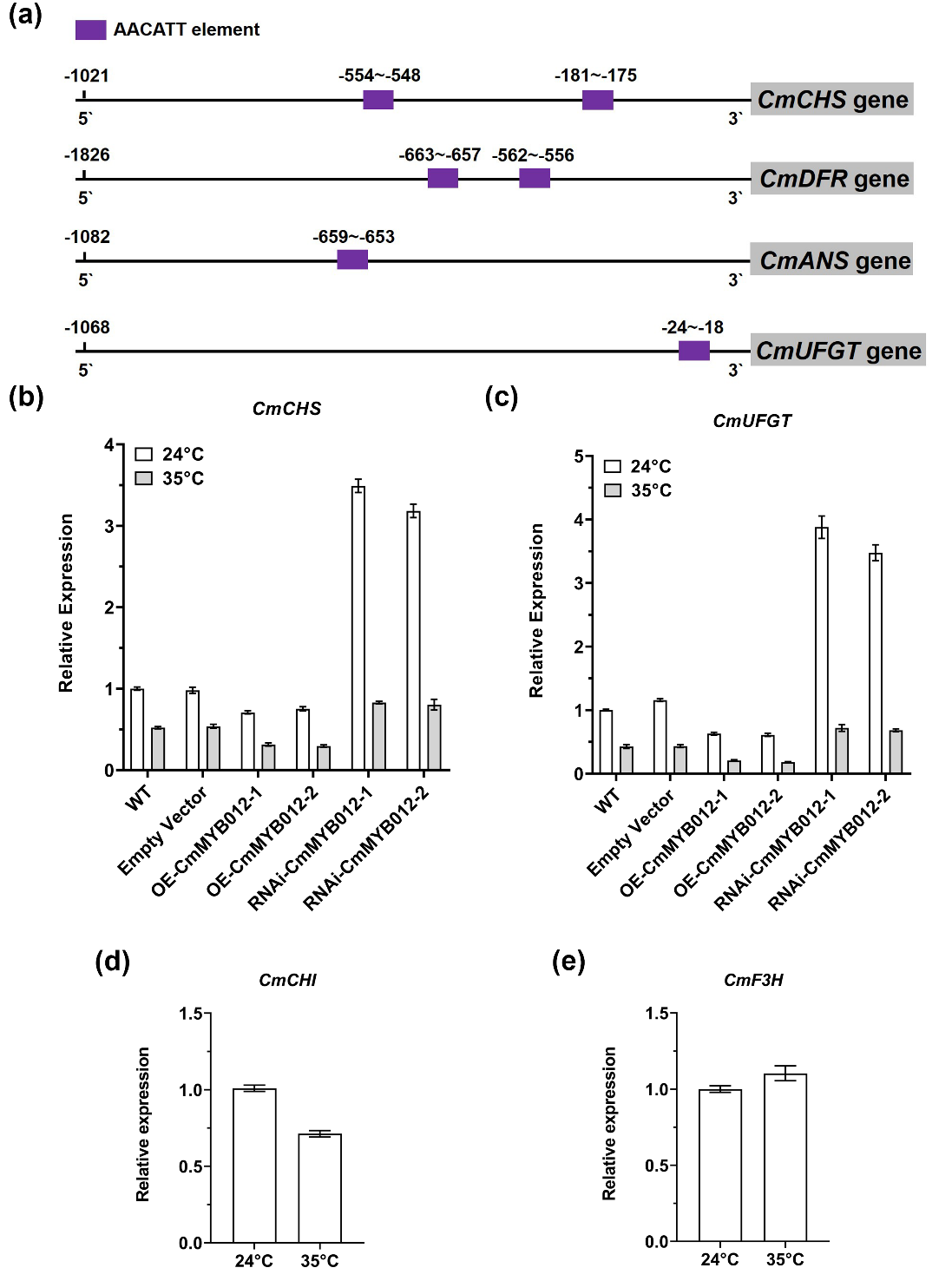


**Supplemental Figure S6. CmMYB012 regulates the expression of *CmCHS* and *CmUFGT***

(a). AACAAT element analysis in the promoters of *CmCHS*, *CmDFR*, *CmANS*, and *CmUFGT*. The purple box represents the AACAAT element. (b) and (c). Relative expression of *CmCHS* and *CmUFGT* in petals of WT and the transgenic plants treated at 24 ℃ and 35 ℃, respectively. The Error bars indicate the SDs for three biological replicates. (d) and (e). Relative expression of *CmCHI* and *CmF3H* in WT plants treated at 24 °C and 35 °C for 6 d, respectively. The Error bars indicate the SDs for three biological replicates.


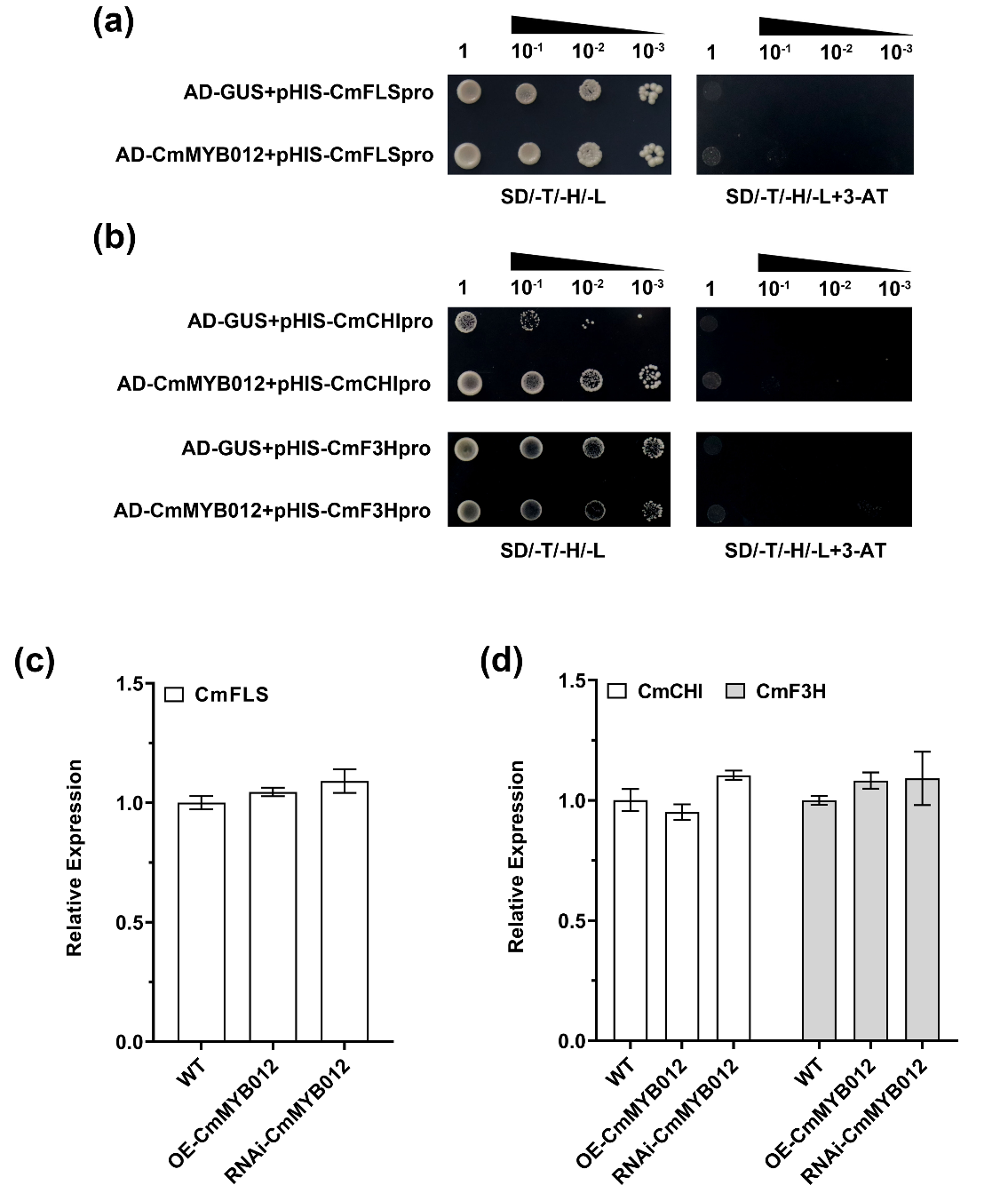


**Supplemental Figure S7. CmMYB012 does not regulate *CmFLS*, *CmCHI*, and *CmF3H* expression in chrysanthemum**

(a). Interaction between CmMYB012 and the promoter of *CmFLS* in yeast cells. SD/-T/-H/-L indicates Trp, His, and Leu dropout synthetic dropout medium. 80 mM 3-AT was used. (b). Interactions among CmMYB012 and the promoters of *CmCHI* and *CmF3H* in yeast cells. SD/-T/-H/-L indicates Trp, His, and Leu dropout synthetic dropout medium. 3-AT concentrations: 50 mM for *CmCHIpro*, 90 mM for *CmF3Hpro*. (c) and (d). Relative expression of *CmFLS*, *CmCHI*, and *CmF3H* in WT and the transgenic plants at 24 °C. The Error bars indicate the SDs for three biological replicates.


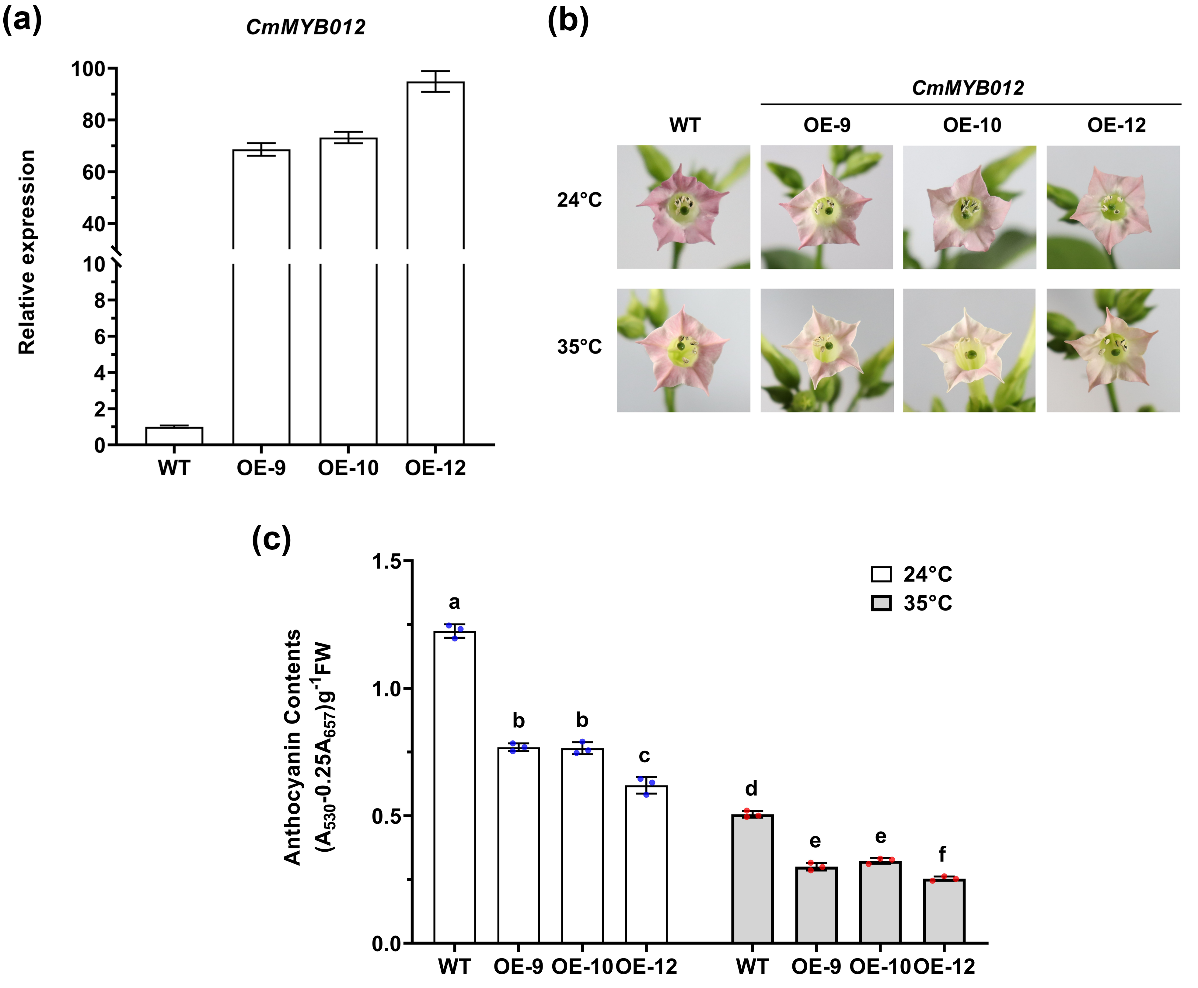


**Supplemental Figure S8. Ectopic expression of *CmMYB012* inhibits anthocyanin biosynthesis in tobacco**

(a). Relative expression of *CmMYB012* in WT and three independent lines of the *OE-CmMYB012* transgenic tobacco plants. The Error bars indicate the SDs for three biological replicates. (b). Flower color phenotypes of WT and the transgenic tobacco plants treated at 24 °C and 35 °C, respectively. (c). Anthocyanin content in WT and the transgenic tobacco plants treated at 24 °C and 35 °C, respectively. The Error bars indicate the SDs for three biological replicates. Samples denoted by different letters are significantly different (p < 0.01, ANOVA, Tukey correction). Points represent each independent measurement.


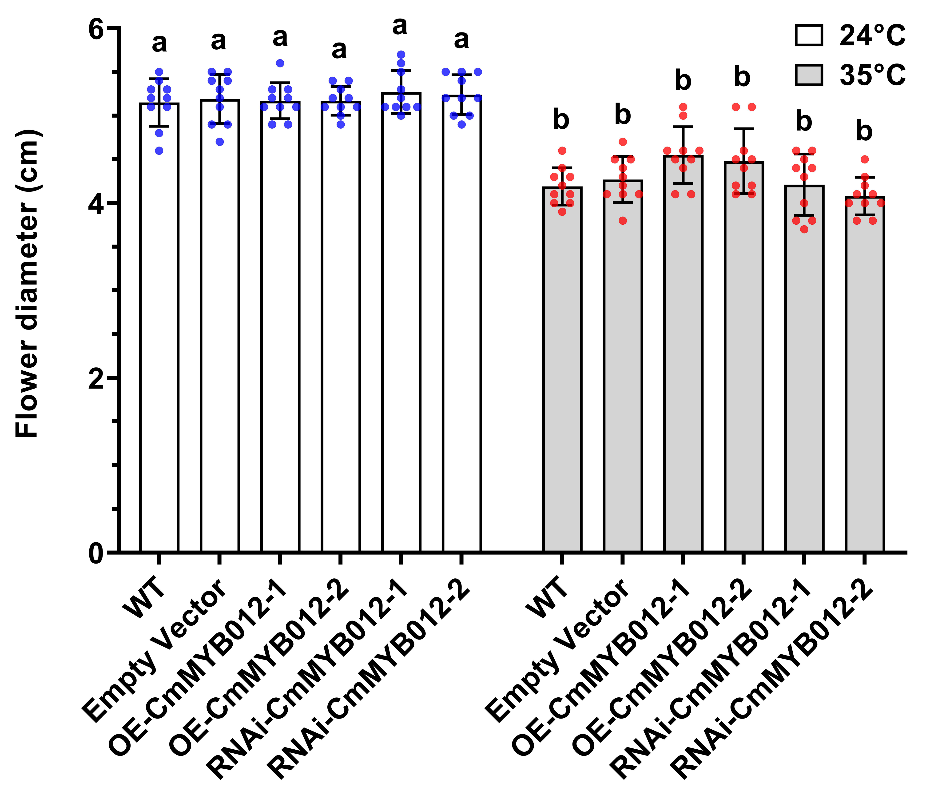


**Supplemental Figure S9. High temperatures inhibit increases in flower diameter in chrysanthemum**

Statistics of flower diameter in WT, the *Empty Vector* (*35S:GFP*), the *OE-CmMYB012* (*35S:CmMYB012-GFP*), and the *RNAi-CmMYB012* transgenic chrysanthemum plants treated at 24°C and 35°C, respectively. WT and the transgenic chrysanthemum plants were treated at 24℃ and 35℃ at budding stage, respectively, and 10 plants of each type were used to randomly select 1 flower to measure the maximum flower diameter after blooming. The Error bars indicate the SDs for ten biological replicates. Samples denoted by different letters are significantly different (p < 0.01, ANOVA, Tukey correction). Points represent each independent measurement.

**Supplemental Table S1**. Primers used for gene cloning

| Primers name | Primers sequence (5’→3’) |
| --- | --- |
| CmMYB012-F | atgggaagagctccttgttgtga |
| CmMYB012-R | tcacagggaccaaccgtgtaa |
| pCold-CmMYB012-F | gagctcggtaccctcgagatgggaagagctccttgt |
| pCold-CmMYB012-R | ctatctagactgcaggtctcacagggaccaaccgt |
| pORE-R4-CmMYB012-F | ccgctcgagatgggaagagctccttgt |
| pORE-R4-CmMYB012-R | tcccccgggcagggaccaaccgtg |
| RNAi-CmMYB012-I | gattggattcgctaatagaccactctctcttttgtattcc |
| RNAi-CmMYB012-II | gagtggtctattagcgaatccaatcaaagagaatcaatga |
| RNAi-CmMYB012-III | gagtagtctattagccaatccattcacaggtcgtgatatg |
| RNAi-CmMYB012-IV | gaatggattggctaatagactactctacatatatattcct |
| RNAi-CmMYB012-A | ctgcaaggcgattaagttgggtaac |
| RNAi-CmMYB012-B | gcggataacaatttcacacaggaaacag |
| pGAD7-CmMYB012-F | cggaattcatgggaagagctccttgt |
| pGAD7-CmMYB012-R | ccctcgagctcacagggaccaaccgtg |
| CmFNSpro-F | agaggagtagcatagggacttaaagtgaca |
| CmFNSpro-R | gttaaagaggttggaaaaaggtgca |
| CmFNSpro-b-F | ggtaacacgttatattctgatcaatat |
| CmFNSpro-b-R | gttaaagaggttggaaaaaggtgca |
| CmFNSpro-c-F | aaaactttgaagtttccattgcat |
| CmFNSpro-c-R | gttaaagaggttggaaaaaggtgca |
| pHIS2-CmFNSpro-F | cggaattcagaggagtagcatagggac |
| pHIS2-CmFNSpro-R | cgacgcgtgttaaagaggttggaaa |
| LUC-CmFNSpro-F | gcgtcgacagaggagtagcatagggac |
| LUC-CmFNSpro-R | cgacgcgtgttaaagaggttggaaa |
| CmCHSpro-F | agcaggatgatgctctattaccaa |
| CmCHSpro-R | cggtgtttaatatcggtgaacgaa |
| CmCHIpro-F | agtccattggtaagagctcttgggctt |
| CmCHIpro-R | ggtagtaagtacagttggggtaaaagaa |
| CmF3Hpro-F | tatcaaagcatataaatggttgtctgt |
| CmF3Hpro-R | tttttattttttcttcacacacttggtg |
| CmFLSpro-F | aatgcacgaaggcacctcactctta |
| CmFLSpro-R | tgtgtgtgcttttgcctcttgttt |
| CmDFRpro-F | gaggctatcaaacggttgtgattca |
| CmDFRpro-R | gttgttttaagcttgtggtttttgaa |
| CmANSpro-F | acaatatgtccaaatttcgaaaaatattga |
| CmANSpro-R | tttgtaagtgttggattttgtggtgtt |
| CmUFGTpro-F | agaggcaattagatgttaccaccgcat |
| CmUFGTpro-R | ttggctgtattttaagaaaatgttgat |

**Supplemental Table S2**. Primers used for RT-qPCR

| Primers name | Primers sequence (5’→3’) |
| --- | --- |
| QM-F | gacgaacagacaacgaaatcaaa |
| QM-R | atagggaggatgatttacgaggt |
| QCS-F | tcggctacagatgggttcaat |
| QCS-R | tttagcacacgaagcccaca |
| QCI-F | cgcaggtgtgagaggtatgga |
| QCI-R | cttaccagcaagcaacggaat |
| QF3-F | cggcctaaggtgccatacaa |
| QF3-R | tcggccctacgtgatttgat |
| QFN-F | gcccagtcctccatcgttac |
| QFN-R | tagcggaccatagcgagttga |
| QFL-F | accactctccatggcgtctt |
| QFL-R | tcccatggttcaccacttga |
| QD-F | gaacccaactaaacccgc |
| QD-R | aatgtgattccgctcctg |
| QA-F | ggattaggacttgaggaggg |
| QA-R | gttgagggcattttgggtag |
| QU-F | tagtggagtcgtgtttggta |
| QU-R | tgaagataggtttttggtgag |
| CmEF1α-F | ttttggtatctggtcctggag |
| CmEF1α-R | ccattcaagcgacagactca |
| CmActin-F | agcttgcatatgttgctcttga |
| CmActin-R | ttaccgtaaaggtccttcctga |

**Supplemental Table S3**. Primers used for ChIP-PCR

| Primers name | Primers sequence (5’→3’) |
| --- | --- |
| P1-F | ggtaacacgttatattctga |
| P1-R | atatacatgcagcggaattaaaac |
| P2-F | gtttattatcgttgttattgtta |
| P2-R | ttttaatttaaaaggcaattgcat |
| P3-F | atgagtgtttaagattcgtctttc |
| P3-R | accttaaagaccaaaaatgcaat |
| P4-F | atggtcaaattgcaggtttcgt |
| P4-R | gacaaatctgcaatttagggac |
| PCK-F | catacaataaaatcttctaaataga |
| PCK-R | aagattaaattgcatttttgtcct |

**Supplemental Table S4**. Detailed information concerning the flavonoids used as standards

| Number | Name | CAS | Retention time (min) | Linear equation | Correlation coefficient(r) |
| --- | --- | --- | --- | --- | --- |
| 1 | Apigenin | 520-36-5 | 16.69 | Y=238.8 + 2831.5*X | 0.9958 |
| 2 | Luteolin | 491-70-3 | 14.83 | Y=845.13 + 1941.6*X | 0.9967 |
| 3 | Kaempferol | 520-18-3 | 16.42 | Y=46.136 + 208.21*X | 0.9952 |
| 4 | Quercetin | 117-39-5 | 13.84 | Y=418.49 + 518.45*X | 0.9901 |
